# HDAC1 acts as a tumor suppressor in ALK-positive anaplastic large cell lymphoma: implications for HDAC inhibitor therapy

**DOI:** 10.1038/s41375-025-02584-9

**Published:** 2025-04-02

**Authors:** Maša Zrimšek, Kristina Draganić, Anna Malzer, Verena Doblmayr, Katarina Mišura, Rafael de Freitas E Silva, Jamie D. Matthews, Fabio Iannelli, Sabrina Wohlhaupter, Carlos Uziel Pérez Malla, Heinz Fischer, Helga Schachner, Ana-Iris Schiefer, Raheleh Sheibani-Tezerji, Roberto Chiarle, Suzanne Dawn Turner, Wilfried Ellmeier, Christian Seiser, Gerda Egger

**Affiliations:** 1 Department of Pathology, Medical University of Vienna, Vienna, Austria; 2 Comprehensive Cancer Center, Medical University of Vienna, Vienna, Austria; 3 Division of Immunobiology, Institute of Immunology, Center for Pathophysiology, Infectiology and Immunology, Medical University of Vienna, Vienna, Austria; 4 Department of Pathology, University of Cambridge, Cambridge, UK; 5 Division of Hematopathology, IEO European Institute of Oncology IRCCS, Milan, Italy; 6 Ludwig Boltzmann Institute Applied Diagnostics, Vienna, Austria; 7 Division of Cell and Developmental Biology, Center for Anatomy and Cell Biology, Medical University of Vienna, Vienna, Austria; 8 Department of Molecular Biotechnology and Health Sciences, University of Torino, Torino, Italy; 9 Department of Pathology, Boston Children’s Hospital and Harvard Medical School, Boston, MA USA; 10 Faculty of Medicine, Masaryk University, Brno, Czech Republic

**Keywords:** T-cell lymphoma, Cancer therapy

## Abstract

Histone deacetylases (HDACs) are frequently deregulated in cancer, and several HDAC inhibitors (HDACi) have gained approval for treating peripheral T cell lymphomas. Here, we investigated the effects of pharmacological or genetic HDAC inhibition on NPM::ALK positive anaplastic large cell lymphoma (ALCL) development to assess the potential use of HDACi for the treatment of this disease. Short-term systemic pharmacological inhibition of HDACs using the HDACi Entinostat in a premalignant ALCL mouse model postponed or even abolished lymphoma development, despite high expression of the NPM::ALK fusion oncogene. To further disentangle the effects of systemic HDAC inhibition from thymocyte intrinsic effects, conditional genetic deletions of HDAC1 and HDAC2 enzymes were employed. In sharp contrast, T cell-specific deletion of *Hdac1* or *Hdac2* in the ALCL mouse model significantly accelerated NPM::ALK-driven lymphomagenesis, with *Hdac1* loss having a more pronounced effect. Integration of gene expression and chromatin accessibility data revealed that *Hdac1* deletion selectively perturbed cell type-specific transcriptional programs, crucial for T cell differentiation and signaling. Moreover, multiple oncogenic signaling pathways, including PDGFRB signaling, were highly upregulated. Our findings underscore the tumor-suppressive function of HDAC1 and HDAC2 in T cells during ALCL development. Nevertheless, systemic pharmacological inhibition of HDACs could still potentially improve current therapeutic outcomes.

## Introduction

Anaplastic large cell lymphoma (ALCL) is a rare, aggressive non-Hodgkin lymphoma of T cell origin, characterized by anaplastic lymphoid cells expressing the CD30 antigen. About 60–80% of ALCL cases harbor a characteristic translocation t(2;5)(p23;q35), resulting in a fusion between the anaplastic lymphoma kinase (*ALK*) and nucleophosmin (*NPM1*) genes [1]. The NPM::ALK fusion oncoprotein is a constitutively active kinase that induces a multitude of downstream signaling pathways ultimately driving malignant transformation of T cells and disease progression [2–4].

Treatment modalities for adult ALCL patients include poly-chemotherapy (CHOP-like regimens) and sometimes radiation of involved sites as front-line therapy [5]. A recent trial showed that the addition of brentuximab vedotin (BV), a CD30 antibody-drug conjugate, to the chemotherapy is a more effective treatment option [6]. For pediatric cases, the chemotherapeutic regimen ALCL99 is used [7]. In 2021 the tyrosine kinase inhibitor Crizotinib was approved for the treatment of relapsed or refractory pediatric ALK-positive (ALK+) ALCL cases [8]. Moreover, results of a recent study demonstrated that the addition of BV and Crizotinib to the ALCL99 as a first-line therapy is beneficial [9, 10]. Unfortunately, therapy resistance to Crizotinib remains a frequent challenge [11–14], necessitating the exploration of additional treatment strategies. Several HDACi, like Belinostat, Romidepsin, and Chidamide, showed favorable clinical efficacy in peripheral T cell lymphomas (PTCLs) and many clinical trials with HDACi in combination with other therapeutic agents are currently ongoing [15].

HDACs are epigenetic enzymes that regulate gene expression by catalyzing the removal of acetyl groups from histones. They are frequently deregulated in hematological malignancies [16]. HDACs can modulate the transcription of oncogenes and tumor suppressor genes, and some HDACs function as the catalytic subunits of multi-protein corepressor complexes, being aberrantly recruited to target genes to drive tumorigenesis [17]. In ALCL, the proapoptotic gene BIM can be epigenetically silenced through the recruitment of the SIN3a corepressor complex, where HDAC1/2 acts as a catalytic core [18]. In addition to histone proteins, HDACs can deacetylate non-histone proteins [19], like STAT3, which is a key signal transmitter in ALCL [4, 20].

Maintaining adequate levels of HDAC1/2 is crucial for normal T cell development, as they are indispensable for preserving the integrity of CD4 lineage T cells by inhibiting RUNX3-CBFβ complexes that can induce CD8 lineage programs in CD4+ T cells [21]. Likewise, dual inactivation of HDAC1/2 in T cells using *Lck-*Cre leads to a developmental blockade, while reduced HDAC activity results in genomic instability and neoplastic transformation [22, 23]. Thus, HDAC1/2 exerts an essential role in maintaining genome stability and the development of mature T cell populations. Consequently, the use of HDAC inhibitors could potentially accelerate lymphomagenesis, especially under certain (pre-malignant) conditions, as demonstrated in a mouse model of acute promyelocytic leukemia [24].

Here we used a murine model of ALCL, driven by the expression of the human fusion oncogene NPM::ALK in T cells, to assess the effects of pharmacological inhibition or genetic deletion of specific class I HDAC isoforms. Systemic administration of HDACi delayed or completely abrogated tumor development, whereas T cell-specific depletion of HDAC1 or HDAC2 or inactivation of the catalytic activity of HDAC1 significantly accelerated lymphomagenesis.

## Methods

### Human samples

The use of archived human FFPE tumor samples was approved by the institutional review board of the Medical University of Vienna (#1224/2014). IHC protocols can be found in Supplementary Methods.

### scRNA-seq

scRNA-seq data were previously published [14], analyses are detailed in Supplementary Methods.

### Mice

*Cd4*-NPM::ALK-transgenic mice [25], loxP-flanked *Hdac1* [26] or *Hdac2* [26] mice, and *Cd4*Cre mice [27] were crossed to obtain NPM::ALK *Hdac1*KO and NPM::ALK *Hdac2*KO mice. Similarly, NPM::ALK *Hdac1*KO mice were crossed with mice with a Rosa26 knock-in (KI) construct containing the *Hdac1* gene with a His141→Ala point mutation, which results in the expression of catalytically inactive HDAC1 [28, 29]. The genetic background of mice was mixed (C57Bl/6xSV/129). The study was approved by the Austrian Federal Ministry for Science and Research (BMWF; GZ.: 66.009/0304.WF/V/3b/2014).

### HDACi treatments and IC50 determination

Mice were treated with HDACi for two consecutive weeks on a five-days-on-two-days-off schedule. Entinostat (Selleckchem) was administered *via* intraperitoneal (IP) injection at indicated concentrations and was diluted in 90% sterile filtered corn oil and 10% DMSO. Human ALCL cell lines were grown from previously established patient-derived xenograft (PDX) models (patient 1: MGS-A-x; patient 2: MTK-A-x; patient 3: GR-ALCL-1) [30]. Details on culturing and IC50 measurements are described in Supplementary Methods.

### Western blotting

Snap-frozen tissues were processed for SDS-PAGE and western blot analysis as previously described [19]. Antibodies and buffers are listed in Supplementary Methods.

### FACS immunophenotyping

Samples were analyzed with a Cytek Aurora cytometer (Cytek Biosciences, Amsterdam, the Netherlands) and quantified using FlowJo^TM^ v10.9.0. Software (BD Life Sciences). Gating strategies are displayed in Supplementary Fig. 4A–C. Protocols and antibodies used are listed in Supplementary Methods.

### ATAC-seq and RNA-seq

Snap-frozen tumor tissue was used from the same tissue. Sample preparation, data processing, and bioinformatics analyses are described in detail in the Supplementary Methods.

## Results

### HDAC inhibitor treatment restricts NPM::ALK-dependent tumor development

**Fig. 1:**
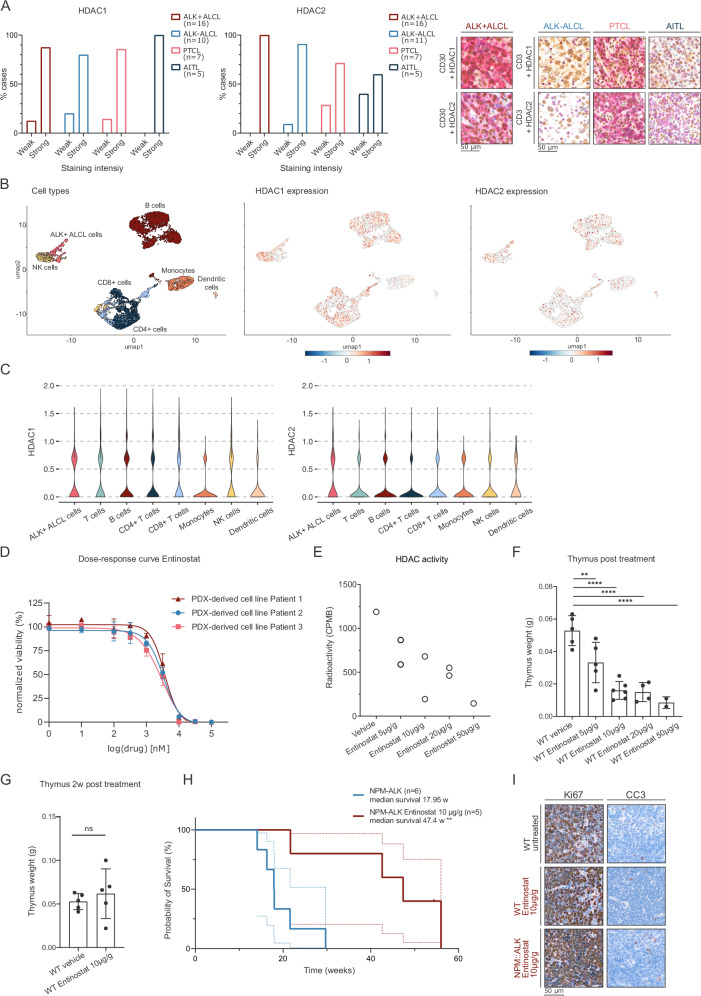
HDAC inhibitor treatment before tumor onset significantly restricts NPM::ALK tumor development in the ALCL mouse model. **A** Bar plots depicting the percentages of HDAC1 (left) or HDAC2 (right) staining intensities (weak, strong) on tissue microarrays (TMAs) containing specified numbers of ALK+ ALCL, ALK− ALCL, PTCL, and AITL patient samples, evaluated by immunohistochemistry (IHC). The right panel displays representative microscopic images of IHC stainings from the TMAs, as described above (scale bar representing 50 μm). Red cytoplasmic/membrane staining represents CD30/CD3 expression, while brown nuclear staining represents HDAC1/HDAC2 expression. Tissues were stained with the corresponding antibodies and counterstained with hematoxylin (blue). **B** UMAP plots of scRNA-seq data from CD45^+^ cells from a primary lymph node of an ALK+ ALCL patient. The left plot shows cells in a dimensional reduction embedding, color-coded according to the different annotated cell types, the middle and right plots show levels of normalized gene expression for *HDAC1* and *HDAC2*. **C** Violin plots depicting normalized expression of *HDAC1* (upper) and *HDAC2* (lower) in color-coded cell types according to (**B**). **D** Dose-response curves of human ALK+ ALCL cell lines, derived from three individual PDX models [30], to Entinostat. Cells were treated for 48 h with a drug concentration range of 0.01 to 100 µM in technical replicates (*n* technical = 3). Each experiment was repeated 3 times (*n* = 3). Dose-response curves were generated with GraphPad Prism 10. Graphs show the mean with standard deviation (SD) for *n* = 3 replicates. **E** HDAC activity levels in thymi of WT mice after 2 weeks of treatment with Entinostat (*n* = 1 biological replicate for vehicle treatment and 50 μg/g/day treatment, *n* = 2 biological replicates for 5 μg/g/day, 10 μg/g/day and 20 μg/g/day treatment, *n* = 2 technical replicates for each biological replicate). Activity levels are measured as counts per minute beta (CPMB). GraphPad Prism version 8.4.3 was utilized for analysis. **F** Thymic weight of WT mice in biological replicates treated for 2 weeks with vehicle (*n* = 5), 5 μg/g/day Entinostat (*n* = 5), 10 μg/g/day Entinostat (*n* = 6), 20 μg/g/day Entinostat (*n* = 4) and 50 μg/g/day Entinostat (*n* = 2). The mean with standard deviation (SD) was plotted using GraphPad Prism version 8.4.3. Statistical significance is indicated by ** for *p* < 0.01 and **** for *p* < 0.0001. **G** Thymic weight of WT mice in biological replicates treated for 2 weeks with vehicle (*n* = 5) or 10 μg/g/day Entinostat (*n* = 5) and then recovered for an additional 2 weeks. Mean with standard deviation (SD) was plotted using GraphPad Prism version 8.4.3. “ns” indicates not significant. **H** Kaplan–Meier survival analysis of NPM::ALK mice (*n* = 6, blue line) and NPM::ALK mice treated with 10 μg/g/day Entinostat (*n* = 5, green line) in biological replicates. The median survival of different genotypes was compared using the Log-rank (Mantel–Cox) test with GraphPad Prism version 8.4.3. Statistical significance is denoted by ** for *p* = 0.0042. **I** Representative microscopic images of Ki67 and CC3 expression based on IHC staining of thymic sections from untreated WT mice, WT mice treated with 10 μg/g/day Entinostat, or NPM::ALK mice treated with 10 μg/g/day Entinostat mice. Thymi were excised immediately after the 2-week treatment period. Sections were counterstained with hematoxylin (blue). The scale bar represents 50 μm.

Using a spectrum of human lymphoma specimens, we observed high levels of HDAC1 and HDAC2 protein expression in the vast majority of ALCL, angioimmunoblastic T cell lymphoma (AITL), and PTCL cases (Fig. 1A). Importantly, PTCL has been previously shown to exhibit aberrant expression of HDACs [31] and HDACi are already approved for its treatment [32, 33]. Interestingly, high HDAC expression was likewise observed in non-malignant T cells for all lymphoma subgroups tested (Supplementary Fig. 1A). To characterize HDAC gene expression levels in different cell types found in ALCL tumors, single-cell RNA-seq data of 2 ALK+ ALCL patients was analyzed from a previously published report [14]. Notably, the expression of HDAC1 and HDAC2 was comparable between ALK+ tumor cells and other immune cells, including T cells, B cells, and NK cells, suggesting that class I HDACs are abundant in normal and malignant immune-cell subsets. (Fig. 1B, C, Supplementary Fig. 1B–D). To investigate the sensitivity of human ALK+ ALCL cells towards HDAC inhibition, we used the class I HDACi Entinostat, which inhibits HDAC1, HDAC2, and HDAC3 (with respective IC50 values of 0.163, 0.396, and 0.605 µM) [34]. We employed three cell lines, established from PDX models of three individual ALCL patients, who were either multi-agent chemotherapy refractory (patient 2) or chemotherapy refractory and crizotinib resistant (patient 1, 3) [30]. All three cell lines showed high sensitivity against Entinostat. Interestingly, similar values were detected both for cell lines established from Crizotinib naïve or refractory PDX models, with IC50 between 3 and 3.6 µM (Fig. 1D).

To study the effects of HDAC inhibition on NPM::ALK transformed cells in more detail, we employed a transgenic NPM::ALK mouse model, which mimics human ALK+ ALCL [25]. First, the effects of a range of HDACi were tested in vitro in primary tumor cell lines isolated from tumors of NPM::ALK mice. Using the pan-HDACi Vorinostat and the class I-specific inhibitor Entinostat, we previously showed high sensitivity of murine ALK+ tumor cell lines, resulting in DNA damage and apoptosis following 48 h of treatment [19]. Moreover, NPM::ALK transformed thymocytes responded to HDACi treatment as indicated by increased core histone acetylation following the 16h-long exposure to Vorinostat, Valproic acid, Entinostat, and Romidepsin at the respective IC50 concentrations (Supplementary Fig. 1E).

Next, pre-tumorigenic NPM::ALK mice were subjected to Entinostat treatment. Dose escalation of Entinostat in WT mice for 2 weeks with 5, 10, 20, and 50 µg Entinostat/g mouse weight/day demonstrated dose-dependent enzyme inhibitory effects as measured by HDAC activity assays in thymocytes (Fig. 1E), accompanied by a dose-dependent acute thymic involution, with full recovery 2 weeks post-treatment cessation (Fig. 1F, G). Due to the toxicity of higher doses, 10 µg/g/day was selected for treatment of NPM::ALK mice, initiated at 6 weeks of age for 2 weeks, to investigate the effects of HDAC inhibition on NPM::ALK lymphomagenesis. Interestingly, HDACi treatment resulted in a notable delay or even prevention of lymphomagenesis, significantly extending the median survival of NPM::ALK-transgenic mice from 17.95 to 47.4 weeks (Fig. 1H). Interestingly, despite the immense decrease in thymus size during the treatment, there was a lack of apoptotic cells and the proliferation of thymocytes remained comparable to untreated counterparts (Fig. 1I). This might indicate that involution could result from a lack of thymic progenitors from the bone marrow to replenish the thymus or rapid clearance of apoptotic cells in the thymus. This raises the question, whether the effects of HDACi treatment are a result of HDAC inhibition in the (pre-)tumor cells, the thymic microenvironment, progenitor compartments, or combinations thereof.

### *Hdac1* loss in T cells accelerates lymphomagenesis

**Fig. 2:**
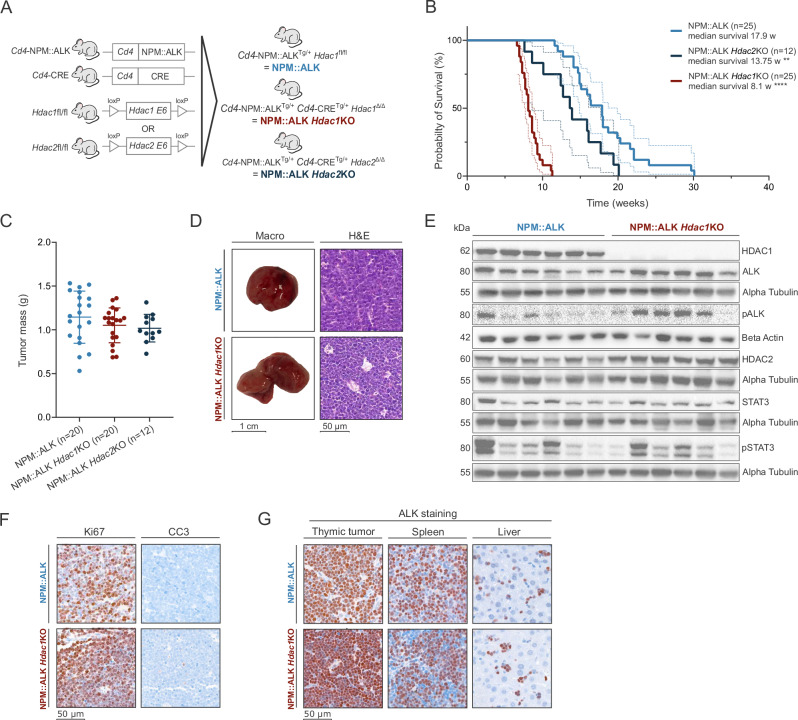
*Hdac1* loss in T cells accelerates lymphomagenesis. **A** Schematic representation of the different mouse models used in the study. The human oncoprotein NPM::ALK was expressed under the T cell-specific *Cd4* promoter. NPM::ALK mice were further crossed with *Cd4*CRE^+^ mice with floxed exons 6 of the *Hdac1* or *Hdac2* gene, which produced T cell-specific NPM::ALK-transgenic mice with additional *Hdac1* or *Hdac2* knockout (NPM::ALK *Hdac1*KO, NPM::ALK *Hdac2*KO). **B** Kaplan–Meier survival analysis of NPM::ALK mice (*n* = 25, light blue line), NPM::ALK *Hdac2*KO mice (*n* = 12, dark blue line), and NPM::ALK *Hdac1*KO mice (*n* = 25, dark red line) in biological replicates. The median survival of different genotypes was compared with the Log-rank (Mantel–Cox) test using GraphPad Prism (version 8.4.3). Statistical significance is indicated by ** for *p* < 0.01 and **** for *p* < 0.0001. **C** Comparison of thymic tumor mass (g) of different genotypes. The mean with standard deviation (SD) is plotted. **D** Representative macroscopic pictures of end-stage thymic tumors (scale bar representing 1 cm) and hematoxylin-eosin (H&E) stained end-stage thymic tumor sections (scale bar representing 50 μm). **E** Immunoblot of protein expression levels of HDAC1, HDAC2, ALK, pALK, STAT3, and pSTAT3 in end-stage thymic tumors excised from NPM::ALK (*n* = 6) and NPM::ALK *Hdac1*KO mice (*n* = 6). Alpha-tubulin or beta-actin were used as loading controls. The numbers on the left indicate the molecular weight of respective proteins in kiloDalton (kDa). **F** Representative microscopic images of Ki67 and CC3 expression analyzed by IHC of end-stage thymic tumor sections (scale bar representing 50 μm). Ki67 was used as a marker of proliferation and CC3 as a marker of apoptosis. Sections were counterstained with hematoxylin (blue). **G** Representative microscopic images of ALK IHC stainings of end-stage thymic tumor, spleen, and liver sections (scale bar representing 50 μm). Sections were counterstained with hematoxylin (blue).

To disentangle the effects of the loss of HDAC activity in pre-tumor cells from the loss in other compartments, *Hdac1* or *Hdac2* were deleted in T cells *via Cd4*Cre in NPM::ALK mice, resulting in NPM::ALK *Hdac1*KO and NPM::ALK *Hdac2*KO mice (Fig. 2A). NPM::ALK-transgenic mice developed thymic tumors with a median survival of 17.9 weeks (Fig. 2B). Surprisingly, deletion of *Hdac1* or *Hdac2* in T cells resulted in strongly accelerated lymphomagenesis with median survivals of 8.1 weeks upon *Hdac1* or 13.75 weeks upon *Hdac2* deletion (Fig. 2B), with tumors of comparable size found in NPM::ALK *Hdac1*KO and NPM::ALK *Hdac2*KO mice (Fig. 2C). Notably, T cell-specific deletion of *Hdac1* in non-ALK-transgenic mice also induced thymic tumors in approximately a quarter of mice at older ages, whereas no signs of malignant transformation were observed in thymi of mice with a deletion of *Hdac2* (Supplementary Fig. 2A).

Due to the stronger effects of HDAC1 loss, we further focused on the NPM::ALK *Hdac1KO* model. Differences in macroscopic tissue architecture were observed, with the NPM::ALK tumors being predominantly round and encapsulated, while NPM::ALK *Hdac1*KO tumors presented the characteristic two-lobed thymus structure (Fig. 2D). HDAC1 loss resulted in a compensatory upregulation of HDAC2 protein and induced ALK kinase activity, as indicated by higher levels of phosphorylated ALK (pALK) (Fig. 2E). pSTAT3, a primary downstream target of ALK, showed a heterogeneous expression pattern, while still corresponding largely to the levels of upstream pALK. No apparent differences in the rate of apoptosis (cleaved caspase 3) or proliferation (Ki67) were observed in end-stage tumors (Fig. 2F). Nearly 100% of cells in tumors of both genotypes expressed high levels of ALK and the ALCL-specific marker CD30 (Fig. 2G, Supplementary Fig. 2B). Disseminated ALK+ cells were detected in spleen and liver tissues of NPM::ALK and NPM::ALK *Hdac1*KO mice (Fig. 2G). Especially in the liver, ALK+ cells were prominent around vessels (Supplementary Fig. 2C), indicating a potential tumor cell dissemination through circulatory and lymphatic systems.

### Accelerated lymphomagenesis depends on the loss of HDAC1 enzymatic activity

**Fig. 3:**
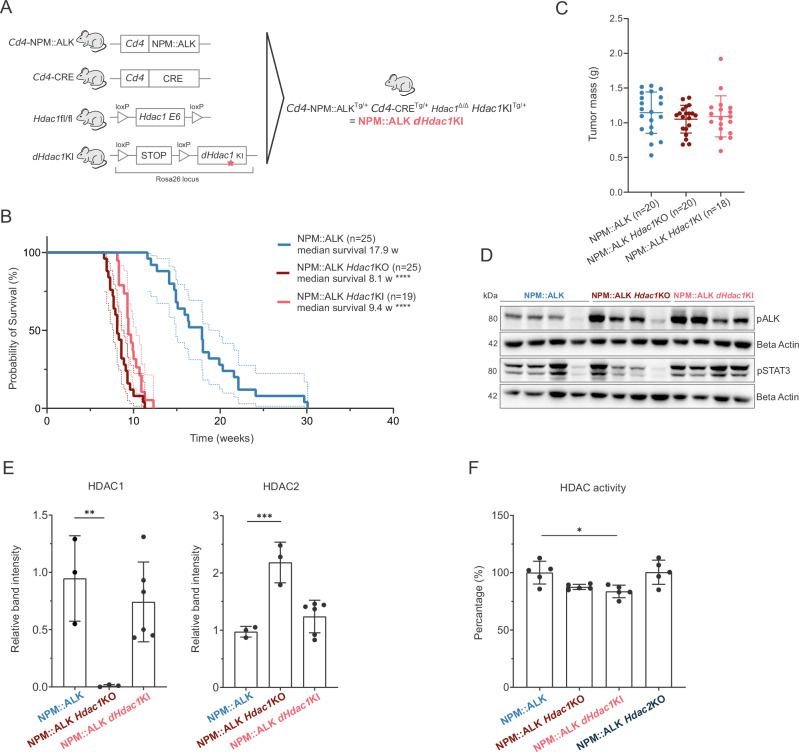
Accelerated lymphomagenesis depends on the loss of HDAC1 enzymatic activity. **A** Schematic representation of mouse models used to generate NPM::ALK mice lacking the endogenous *Hdac1* gene but expressing a catalytically dead, mutated HDAC1 protein (dHDAC1) that is unable to deacetylate proteins (NPM::ALK *dHdac1*KI mice). *Cd4* NPM::ALK mice were crossed with CRE^+^ mice with floxed exons 6 of *Hdac1* to obtain NPM::ALK mice with *Hdac1*KO. These were further crossed with mice containing the *dHdac1* gene inserted into Rosa26 locus together with a floxed stop cassette. **B** Kaplan–Meier survival analysis of NPM::ALK mice (*n* = 25, blue line), NPM::ALK *dHdac1*KI mice (*n* = 19, pink line), and NPM::ALK *Hdac1*KO mice (*n* = 25, red line) in biological replicates. The median survival of different genotypes was compared using the Log-Rank (Mantel–Cox) test with GraphPad Prism (version 8.4.3). Statistical significance is indicated by **** for *p* < 0.0001. **C** Comparison of thymic tumor mass (g) of different genotypes. The mean with standard deviation (SD) is plotted. **D** Immunoblot showing protein levels of pALK and pSTAT3 in end-stage thymic tumors isolated from NPM::ALK (*n* = 4), NPM::ALK *Hdac1*KO (*n* = 4) and NPM::ALK *Hdac1*KI (*n* = 4) mice. Beta-actin was used as a loading control. The numbers on the left indicate the molecular weight of analyzed proteins in kiloDalton (kDa). **E** Quantification of HDAC1 (left) and HDAC2 (right) protein levels in end-stage thymic tumors of different genotypes from immunoblots shown in Supplementary Fig. 3. The mean with standard deviation (SD) is plotted. HDAC1 and HDAC2 protein levels were normalized according to beta-actin used as a loading control. Groups were compared using one-way ANOVA corrected for multiple comparisons with GraphPad Prism version 8.4.3. Statistical significance is indicated by ** for *p* < 0.01 and *** for *p* < 0.001. **F** HDAC activity levels were measured in end-stage thymic tumors of different genotypes (*n* = 5 biological replicates and *n* = 2 technical replicates were used for each genotype). Counts per minute beta (CPMB) values corresponding to HDAC activity levels were converted to percentages (%) with average NPM-ALK tumor HDAC activity set at 100%. The mean with standard deviation (SD) is plotted. Groups were compared using one-way ANOVA corrected for multiple comparisons using GraphPad Prism version 8.4.3. Statistical significance is indicated by * for *p* < 0.05.

HDAC1 is part of multi-protein corepressor complexes [35]. Thus, besides its enzymatic function, HDAC1 is also required for complex formation. To unravel its catalytic and scaffolding functions, we utilized *dHdac1* knock-in (KI) mice that express a catalytically inactive (dead) HDAC1 protein, which can still integrate into corepressor complexes [28, 29]. This model closer reflects the effects of HDACi, which are small molecules binding to the catalytic pocket of HDAC proteins [36]. The *dHdac1*KI was bred into NPM::ALK *Hdac1*KO mice, generating offspring only expressing catalytically inactive HDAC1 (Fig. 3A). Similar to NPM::ALK *Hdac1*KO mice, NPM::ALK *dHdac1*KI mice exhibited highly accelerated lymphomagenesis and developed thymic tumors with a median survival of 9.4 weeks (Fig. 3B). This was evident through loss of the typical thymus architecture of NPM::ALK *Hdac1*KO/KI mice within 3–4 weeks, compared to at least 10 weeks for NPM::ALK mice and reflected in the thymus weight of mice of different genotypes depending on their age (Supplementary Fig. 3A, B). The size of end-stage tumors was comparable among all genotypes (Fig. 3C). The *dHdac1*KI alone was not sufficient to induce thymic lymphomas (Supplementary Fig. 3C). Moreover, hyperactivation of the ALK kinase, as indicated by higher levels of pALK, was observed in NPM::ALK *Hdac1*KI tumors, associated also with high pSTAT3 protein levels for most of the HDAC1 inactive tumors (Fig. 3D). Of note, the loss of HDAC1 catalytic activity did not induce a significant upregulation of HDAC2, as observed in the NPM::ALK *Hdac1*KO (Fig. 3E, Supplementary Fig. 3D). Further, we performed HDAC activity assays to measure the overall enzymatic activity of HDACs in tumors of different genotypes. The activity of NPM::ALK *dHdac1*KI samples was significantly lower as compared with NPM::ALK samples (average reduction of 16.4%) (Fig. 3F). *Hdac1*KO samples showed a smaller reduction in overall HDAC activity (average reduction of 12,4%) with borderline significance compared to NPM::ALK tumors (*p* = 0.059), reflecting the compensatory function of HDAC2.

Together, these data suggest that the loss of HDAC1 enzyme activity is the major factor for accelerated lymphomagenesis and that both HDAC complex formation and HDAC2 activity are less relevant for this process.

### Loss of HDAC1 protein or HDAC1 catalytic activity causes changes in the immunophenotype

**Fig. 4:**
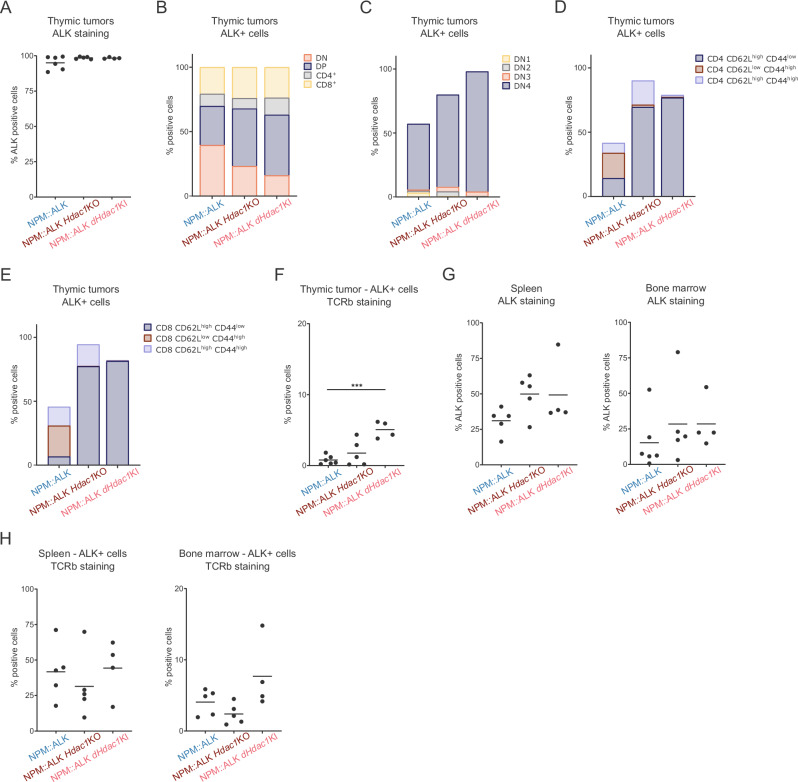
Loss of HDAC1 protein or HDAC1 catalytic activity causes changes in the immunophenotype. **A** Percentage of ALK+ cells in end-stage thymic tumors of different genotypes (NPM::ALK *n* = 6, NPM::ALK *Hdac1*KO *n* = 5, and NPM::ALK *Hdac1*KI *n* = 4) assessed by FACS. The horizontal line represents the average percentage of positive cells of biological replicates for each genotype. **B** Average distribution of markers for double negative (DN; CD4^-^CD8^-^), double positive (DP; CD4^+^CD8^+^), CD4^+^ and CD8^+^ cells among ALK+ cells in end-stage thymic tumors of indicated genotypes: NPM::ALK *n* = 6, NPM::ALK *Hdac1*KO *n *= 5 and NPM::ALK *Hdac1*KI *n* = 4. **C** Average percentage of ALK+ cells exhibiting features of double negative 1 (DN1), double negative 2 (DN2), double negative 3 (DN3), and double negative 4 (DN4) cells in end-stage thymic tumors of different genotypes: NPM::ALK *n* = 6, NPM::ALK *Hdac1*KO *n* = 5 and NPM::ALK *Hdac1*KI *n* = 4. **D** The average percentage of ALK+ cells exhibiting features of CD4 naïve (CD4^+^ CD62L^high^ CD44^low^), CD4 effector (CD4^+^ CD62L^low^ CD44^high^) or CD4 central memory T cells (CD4^+^ CD62L^high^ CD44^high^) in end-stage thymic tumors of different genotypes: NPM::ALK *n* = 6, NPM::ALK *Hdac1*KO *n* = 5 and NPM::ALK *Hdac1*KI *n* = 4. **E** Average percentage of ALK+ cells exhibiting features of CD8 naïve (CD8^+^ CD62L^high^ CD44^low^), CD8 effector (CD8^+^ CD62L^low^ CD44^high^) or CD8 central memory T cells (CD8^+^ CD62L^high^ CD44^high^) in end-stage thymic tumors of different genotypes: NPM::ALK *n* = 6, NPM::ALK *Hdac1*KO *n* = 5 and NPM::ALK *Hdac1*KI *n* = 4. **F** Percentage of ALK+ cells expressing TCRb in end-stage thymic tumors of different genotypes. The horizontal line represents the average percentage of positive cells of biological replicates for each genotype. Groups were compared using one-way ANOVA corrected for multiple comparisons using GraphPad Prism version 8.4.3. Statistical significance is indicated by *** for *p* < 0.001. **G** Percentage of ALK+ cells in the spleen (left) and bone marrow (right) isolated from mice of different genotypes presented with end-stage thymic tumors. The horizontal line represents the average percentage of positive cells of biological replicates for each genotype: NPM::ALK *n* = 5 (left) and NPM::ALK *n* = 6 (right), NPM::ALK *Hdac1*KO *n* = 5 and NPM::ALK *Hdac1*KI *n* = 4. **H** Percentage of ALK+ cells expressing TCRb in the spleen (left) and bone marrow (right) isolated from mice of different genotypes presented with end-stage thymic tumors. The horizontal line represents the average percentage of positive cells of biological replicates for each genotype: NPM::ALK *n* = 5, NPM::ALK *Hdac1*KO *n* = 5, and NPM::ALK *Hdac1*KI *n* = 4.

To evaluate potential immunophenotypic alterations of HDAC1-depleted tumors, we employed multi-parametric FACS analysis for 19 different immune-cell markers on end-stage tumors, spleens, and bone marrow isolated from NPM::ALK, NPM::ALK *Hdac1*KO, and NPM::ALK *dHdac1*KI mice. Unsupervised clustering (tSNE) of CD45^+^ cells showed a clear separation of NPM::ALK tumors from NPM::ALK *Hdac1*KO and NPM::ALK *dHdac1*KI tumors (Supplementary Fig. 4D). Intracellular staining of tumors for ALK expression revealed that the vast majority of cells were ALK+ for all three genotypes (Fig. 4A, Supplementary Fig. 4E). The analysis of the thymocyte population based on CD4 and CD8 expression showed that NPM::ALK *Hdac1*KO and NPM::ALK *dHdac1*KI tumors exhibited more CD4^+^CD8^+^ double positive (DP) cells, while NPM::ALK tumors showed the highest proportion of cells in the double negative (DN) stage (Fig. 4B). DN cells in all tumors appeared predominantly in the CD44^−^CD25^−^ DN4 stage (Fig. 4C). We further assessed whether ALK+ tumor cells expressing CD4 or CD8 also expressed CD44 or CD62L markers, usually used to distinguish naïve (CD62L^high^ CD44^low^), effector (CD62L^low^ CD44^high^) or central memory T cells (CD62L^high^ CD44^high^). While ALK+ cells from NPM::ALK tumors exhibited a considerable heterogeneity, ALK+ cells from NPM::ALK *Hdac1*KO and NPM::ALK *dHdac1*KI tumors mostly resembled a naïve CD4 or CD8 phenotype (CD62L^high^ CD44^low^) (Fig. 4D, E). Consistent with this, the expression of CD62L, a homing receptor for secondary lymphoid organs, separated NPM::ALK from NPM::ALK *Hdac1*KO and NPM::ALK *dHdac1*KI tumors in the unsupervised clustering analysis (Supplementary Fig. 4F). While NPM::ALK transformed T cells showed very low levels of TCRb expression, as previously shown [37], ALK+ cells from NPM::ALK *Hdac1*KO and NPM::ALK *dHdac1*KI tumors exhibited a higher frequency of TCRb+ cells in their thymic tumors (Fig. 4F). The NPM::ALK mouse model develops lymphoma with thymic and not peripheral presentation, like observed in human disease. To date, only RAG-competent NPM::ALK/OT1 mice developed peripheral disease and authors hypothesized that NPM::ALK transformed cells require transient TCR expression for thymic egress [38]. In line with this, we observed a trend of more successful ALK+ cell colonization from thymic tumors to spleen and bone marrow of NPM::ALK *Hdac1*KO and NPM::ALK *dHdac1*KI compared to NPM::ALK mice (Fig. 4G), paralleled by elevated levels of TCRb expression of ALK+ cells found in spleens and bone marrow from mice lacking HDAC1 protein or enzymatic activity (Fig. 4H). Together, these data suggest that loss of HDAC1 protein and activity results in a shift of the immunophenotype of ALK+ tumor cells, with higher numbers of cells expressing the TCR, seemingly facilitating increased tumor cell dissemination.

### Loss of *Hdac1* selectively perturbs cell-type-specific transcription

**Fig. 5:**
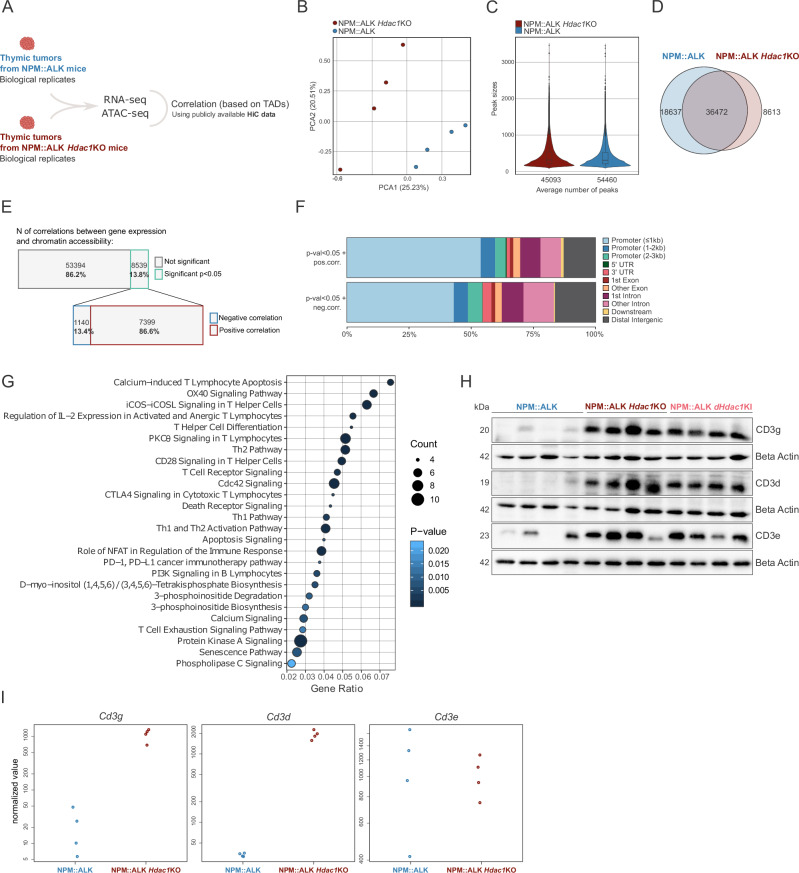
Loss of *Hdac1* selectively perturbs cell-type-specific transcription. **A** Schematic representation of ATAC- and RNA-seq experiments. ATAC- and RNA-seq were performed in parallel using end-stage thymic tumors from NPM::ALK mice (biological replicates *n* = 4, blue) and NPM::ALK *Hdac1*KO mice (biological replicates *n* = 4, red). ATAC- and RNA-seq were correlated with topologically associating domains (TADs) inferred from publicly available HiC data [40]. **B** Principal Component Analysis (PCA) of ATAC-seq data illustrating the similarity/variance of NPM::ALK (blue) and NPM::ALK *Hdac1*KO (red) samples. **C** Violin plot showing the distribution of peak sizes and the average number of peaks in NPM::ALK (blue) and NPM::ALK *Hdac1*KO (red) samples based on ATAC-seq analyses. **D** Venn diagram depicting shared and unique open chromatin regions (=peaks) between NPM::ALK (blue) and NPM::ALK *Hdac1*KO (red) samples. **E** Bar charts representing the number and percentages of overall and statistically significant (*p* < 0.05) correlations between RNA- and ATAC-seq data (upper), as well as the number and percentage of negative and positive correlations among the statistically significant (*p* < 0.05) correlations (lower). **F** Stacked bar chart representing the percentage of open chromatin regions located in different genomic regions, according to the legend on the right side, comparing the open chromatin regions that are positively correlated (*p* < 0.05) with changes in gene expression (e.g., open chromatin in promoter region leads to higher gene expression) (upper) and open chromatin regions that are negatively correlated (*p* < 0.05) with changes in gene expression (e.g., open chromatin in promoter region leads to lower gene expression) (lower). **G** Bubble chart representing significantly enriched pathways in NPM::ALK *Hdac1*KO end-stage thymic tumors as compared to NPM::ALK end-stage thymic tumors based on Ingenuity Pathway Analysis (IPA®) of upregulated genes (RNA-seq: |LFC| ≥ 1, adj *p* < 0.05) that were correlated with changes in chromatin accessibility (correlation *p* < 0.5). The size of the circles represents the number of genes affected within a given pathway, the color indicates the significance level based on the gradient scheme on the right. **H** Immunoblot showing protein levels of CD3d, CD3g and CD3e in end-stage thymic tumors excised from NPM::ALK (*n* = 4), NPM::ALK *Hdac1*KO (*n* = 4) and NPM::ALK *Hdac1*KI mice (*n* = 4). Beta-actin was used as a loading control. The numbers on the left indicate the molecular weight of analyzed proteins in kiloDalton (kDa). **I** VST (variance stabilizing transformation) normalized counts based on RNA-seq analysis for *Cd3g*, *Cd3d*, and *Cd3e* comparing NPM::ALK (blue) and NPM::ALK *Hdac1*KO (red) samples.

To further evaluate the consequences of *Hdac1* loss on chromatin architecture and gene expression, we focused on end-stage NPM::ALK and NPM::ALK *Hdac1*KO tumors and performed parallel ATAC- and RNA-sequencing on biological replicates of both genotypes (Fig. 5A). Principal component analysis revealed a clear separation of the two groups based on their accessible chromatin regions (Fig. 5B). Notably, *Hdac1* loss did not result in stochastic or global chromatin opening, indicated by comparable numbers and sizes of peaks representing accessible chromatin (Fig. 5C), and similar nuclear structures and chromatin architecture visualized by transmission electron microscopy (Supplementary Fig. 5A). In addition to 36 472 overlapping peaks between the two groups, genotype-specific accessible regions were identified, encompassing 18 736 in NPM::ALK and 8 613 unique peaks in NPM::ALK *Hdac1*KO tumors (Fig. 5D). RNA-seq analyses of the same tumors revealed 785 up- and 723 downregulated genes between the two groups (adj *p* < 0.05 and |LFC| ≥ 1) (Supplementary Fig. 5B).

Next, ATAC-seq data were integrated with RNA-seq data to discern functional promoters/enhancers associated with alterations in chromatin accessibility and dysregulated transcription. It was previously shown, that linear proximity cannot be the only point of reference when correlating enhancers with their potential target genes and that it is necessary to consider long-range promoter/enhancer interactions [39]. Thus, publicly available chromosome conformation capture (HiC) data of T cells were used to delineate cell-type-specific topologically associated domains (TADs) [40]. Correlations between open chromatin regions in proximal and distal regulatory elements and transcriptionally active genes were then inferred within the boundaries of every TAD, identifying a total of 8539 significant correlations (*p* < 0.05). Among these, 7399 represented positive correlations, signifying that chromatin opening in promoter/enhancer regions corresponded to higher gene expression, while 1140 displayed negative correlations, where open chromatin regions corresponded to decreased expression (Fig. 5E). Peaks associated with up- or downregulated expression did not differ in their size (data not shown), however, positively correlated peaks were more frequently observed in promoter regions as compared to negatively correlated ones (Fig. 5F).

Ingenuity Pathway Analysis (IPA®) [41] of upregulated genes (adj *p* < 0.05 and |LFC| ≥ 1) with changed chromatin accessibility (correlation *p* < 0.05) in NPM::ALK *Hdac1*KO tumors revealed that the loss of *Hdac1* selectively perturbed cell-type-specific transcription, with top upregulated genes implicated in T cell activation and pathways critical for T cell proliferation and survival such as the OX40 signaling, iCOS-iCOSL signaling or IL-2 signaling (Fig. 5G, Supplementary Table 1). These pathways were previously associated with ALCL or T cell lymphoma subtypes [42–44] and might be induced by increased ALK signaling in NPM::ALK *Hdac1*KO tumors. Of note, increased expression levels of *Cd4* and *Cd8* (Cd4: LFC = 2.27, adj *p* = 4.90E-04; Cd8: LFC = 3.6, adj *p* = 2.40E-02) were observed, in line with the increase in DP thymocytes in NPM::ALK *Hdac1*KO tumors seen in immunophenotyping (Fig. 4B). Furthermore, a marked upregulation of the CD3d/g/e TCR co-receptor was detected in NPM::ALK *Hdac1*KO tumors on protein levels (Fig. 5H) and upregulation of CD3d/g on mRNA level (Fig. 5I), consistent with ATAC-seq data showing increased chromatin accessibility in the CD3g/d promoter regions (Supplementary Fig. 5C). The upregulation of CD3d/g/e proteins was further confirmed in NPM::ALK *Hdac1*KI tumors, mimicking the NPM::ALK *Hdac1*KO tumors (Fig. 5H).

Together, these findings underline the essential function of HDAC1 for the maintenance of T lineage-specific gene expression programs and suggest that the TCR and its co-receptors, which are usually silenced in ALCL [37, 45], remain active upon HDAC1 depletion.

### Loss of *Hdac1* hyperactivates oncogenic transcription

**Fig. 6:**
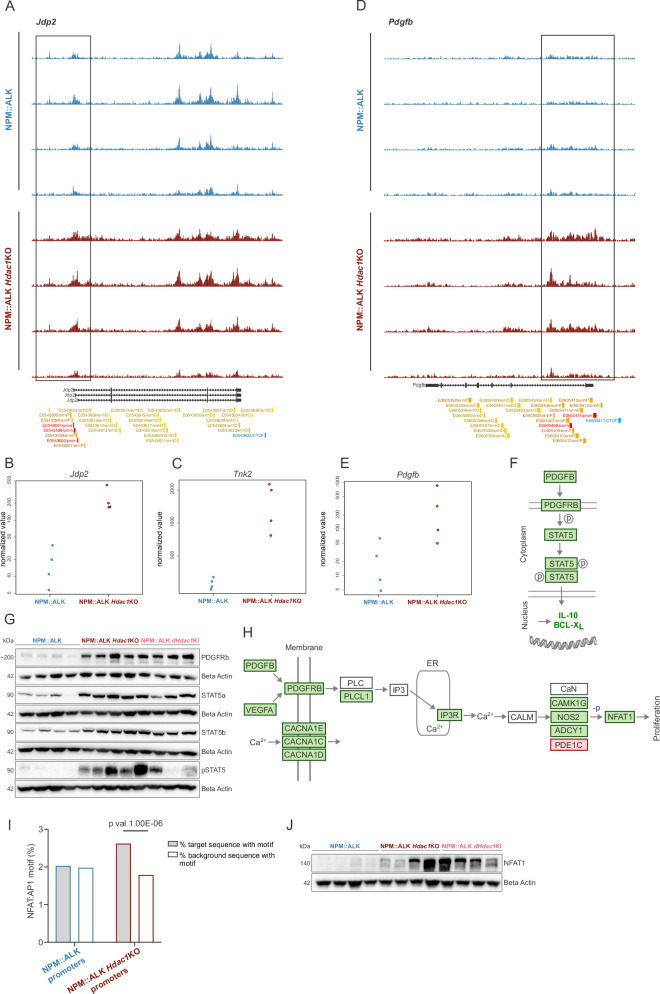
Loss of *Hdac1* hyperactivates oncogenic transcription. **A** ATAC-seq tracks downloaded from the UCSC Genome Browser [65] depicting peaks, which represent chromatin accessibility in the *Jdp2* gene. Biological replicates of NPM::ALK end-stage thymic tumors (*n* = 4, blue) and of NPM::ALK *Hdac1*KO end-stage thymic tumors (*n* = 4, red) are shown. The Gencode track (Gencode VM23 release) is displayed below the ATAC-seq tracks, indicating different transcripts of the *Jdp2* gene. Colored boxes on the bottom show ENCODE candidate Cis-Regulatory Elements (cCREs) combined from all available cell types (red promoter, orange proximal enhancer, yellow distal enhancer, blue CTCF binding sites). **B** VST normalized counts based on RNA-seq analysis for *Jdp2* comparing NPM::ALK (blue) and NPM::ALK *Hdac1*KO (red) samples. **C** VST normalized counts based on RNA-seq analysis for *Tnk2* comparing NPM::ALK (blue) and NPM::ALK *Hdac1*KO (red) samples. **D** ATAC-seq tracks for the *Pdgfb* gene as in **A**. **E** VST normalized counts based on RNA-seq analysis for *Pdgfb* comparing NPM::ALK (blue) and NPM::ALK *Hdac1*KO (red) samples. **F** Schematic representation of the PDGFRB/STAT5 signaling pathway. Green boxes indicate genes upregulated in NPM::ALK *Hdac1*KO tumors as compared to NPM::ALK tumors based on RNA-seq data. **G** Immunoblot showing protein levels of PDGFRb, STAT5a, STAT5b and pSTAT5 in end-stage thymic tumors excised from NPM::ALK (*n* = 4), NPM::ALK *Hdac1*KO (*n* = 4) and NPM::ALK *Hdac1*KI mice (*n* = 4). Beta-actin was used as a loading control. The numbers on the left indicate the molecular weight of analyzed proteins in kiloDalton (kDa). **H** Schematic representation of the Ca^2+^ signaling pathway. Green boxes indicate genes upregulated, red boxes indicate genes downregulated in NPM::ALK *Hdac1*KO tumors as compared to NPM::ALK tumors based on RNA-seq data. ER, *endoplasmic reticulum*. **I** Bar plot depicting the results of the Homer Motif analysis [66], indicating enrichment of the NFAT:AP1 motif in promoter peaks of NPM::ALK *Hdac1*KO samples (red) compared to NPM::ALK samples (blue) or control sequences (background) identified by ATAC-seq analysis. **J** Immunoblot showing protein levels of NFAT1 in end-stage thymic tumors excised from NPM::ALK (*n* = 4), NPM::ALK *Hdac1*KO (*n* = 4) and NPM::ALK *Hdac1*KI mice (*n* = 4). Beta-actin was used as a loading control. The numbers on the left indicate the molecular weight of analyzed proteins in kiloDalton (kDa).

To delineate the molecular mechanisms driving accelerated lymphomagenesis, we scrutinized chromatin and transcriptional alterations in previously identified HDAC1 and NPM::ALK target genes. The MYC oncogene, a central factor for ALK-driven lymphomagenesis [46, 47], showed comparably high chromatin accessibility in its promoter/enhancer regions as well as mRNA and protein expression in NPM::ALK and NPM::ALK *Hdac1*KO tumors (Supplementary Fig. 6A–C). NPM::ALK *Hdac1*KO tumors displayed augmented promoter accessibility (LFC = 1.52, *p* = 1.93E-4) (Fig. 6A) and gene expression (LFC = 3.76, adj *p* = 9.5E-10) of the *Jpd2* gene (Fig. 6B), a MYC-collaborating and p53-suppressing factor previously shown to be upregulated in T cell lymphomas that developed as a consequence of loss of HDAC activity [23]. Furthermore, the oncogenic kinase gene *Tnk2*, implicated in cell survival, proliferation, and migration was upregulated in NPM::ALK *Hdac1*KO tumors (LFC = 2.43, adj *p* = 2.50E-10) (Fig. 6C), which potentially enhanced the NPM::ALK oncogenic cascade *via* interaction with NPM::ALK and co-activation of STAT signaling [48].

Importantly, we found an upregulation of the PDGFRB-STAT5-IL10 oncogenic axis, which was recently shown to be crucial for the aggressiveness of ALK+ ALCL [49]. In NPM::ALK *Hdac1*KO tumors, chromatin accessibility in the promoter region of the *Pdgfb* gene was highly increased (LFC = 9.4, *p* = 1.69E-4) (Fig. 6D), concomitant with a significant upregulation of *Pdgfb* mRNA (LFC = 3.69, adj *p* = 1.80E-02) (Fig. 6E). Moreover, increased gene expression of *Vegfa, Pdgfrb, Stat5a*, *Il10* and *Bcl-xL* was observed in NPM::ALK *Hdac1*KO tumors (|LFC| ≥ 1, adj *p* < 0.05) (Fig. 6F, Supplementary Fig. 6D). The hyperactivation of the PDGFRB-STAT5 axis was corroborated at the protein level in biological replicates of NPM::ALK *Hdac1*KO tumors, demonstrating consistent upregulation of PDGFRB, STAT5A/B and phosphorylation of total STAT5 (Fig. 6G). Moreover, increased ALK activity and upregulation of the PDGFRB-STAT5-IL10 oncogenic axis were confirmed in NPM::ALK *Hdac1*KI tumors, showcasing that their deregulation likely depends on the catalytic activity of HDAC1.

The PDGFRB is implicated in multiple pathways and its activation can also lead to the release of Ca^2+^ from the *endoplasmic reticulum* (ER). Furthermore, it was shown that NPM::ALK can mimic TCR signaling, mostly *via* the oncogenic Ras pathway, but it is also weakly coupled to the calcium/NFAT pathway [50]. Notably, calcium signaling was among the top significantly enriched pathways identified in NPM::ALK *Hdac1*KO tumors based on gene expression data (Fig. 5G, Supplementary Table 1). Several components of the calcium pathway, including *Plcl1*, *Itpr3*, *Camk1g*, *Nos2*, *Adcy1*, as well as the TF *Nfat1* were significantly upregulated (|LFC | ≥ 1, adj *p* < 0.05) (Fig. 6H, Supplementary Fig. 6E). Calcium-dependent NFAT TFs can act synergistically with AP1 TFs [51], which are known to be aberrantly expressed in ALK+ ALCL [52]. Accordingly, we further examined TF motifs in open chromatin regions in NPM::ALK and NPM::ALK *Hdac1*KO tumors. The NFAT:AP1 motif was significantly overrepresented (*p* = 1.00E-06) compared to background in NPM::ALK *Hdac1*KO tumors but not in NPM::ALK tumors (Fig. 6I). NFAT proteins are furthermore key regulators of T cell development [53] and could help explain the deregulation of T cell-specific pathways (Fig. 5G). Upregulation of NFAT1 in NPM::ALK *Hdac1*KO tumors as well as in NPM::ALK *Hdac1*KI tumors was further confirmed on the protein level (Fig. 6J). Upon prolonged Ca^2+^ signaling, ER Ca^2+^ can become depleted and extracellular Ca^2+^ influx is initiated to maintain the signaling. Along these lines, we observed a significant upregulation of the calcium channel encoding genes *Cacna1e, Cacna1c*, and *Cancna1d* in NPM::ALK *Hdac1*KO tumors (|LFC| ≥ 1, adj *p* < 0.05) (Fig. 6H, Supplementary Fig. 6F).

All in all, loss of *Hdac1* in T cells results in hyperactivation of pro-oncogenic transcription programs, suggesting that the accelerated lymphomagenesis is likely a consequence of synergistic effects of multiple deregulated pathways, with a strong involvement of PDGRFB- and Ca^2+^ signaling. Moreover, the accelerated lymphomagenesis and deregulation of oncogenic pathways in NPM::ALK-transgenic mice was highly dependent on the catalytic activity of HDAC1, since the same pathways were consistently found to be deregulated in NPM::ALK *Hdac1*KI tumors.

## Discussion

Our study contributes novel insights into the tumor-suppressive roles of HDACs in the context of T cell lymphoma. We find that T cell-specific deletion of *Hdac1* or *Hdac2* drastically accelerates NPM::ALK-driven lymphomagenesis, with a more pronounced effect observed upon HDAC1 loss. This finding suggests a distinct contribution of HDAC1 and HDAC2 loss to the transformation of T cells, despite their high sequence homology. Interestingly, pharmacological inhibition of HDACs using Entinostat yielded contrasting results compared to genetic loss of HDAC1 protein or enzymatic activity. Entinostat treatment significantly delayed or even prevented tumor development in pre-tumorigenic mice, despite the persistent activity of NPM::ALK signaling. This discrepancy needs to be further evaluated, but might be explained by the following reasons. Firstly, Entinostat as a class I-specific HDACi inhibits HDAC1 as well as HDAC2 and HDAC3, while in the case of genetic loss of *Hdac1*, HDAC2 and HDAC3 remain expressed. Similarly, complete loss of HDAC1 and HDAC2 in thymocytes results in a block in T cell development, while gradual loss of HDAC activity induces lymphoblastic lymphoma [23]. Furthermore, the observed acute thymic involution following Entinostat treatment, also described in the non-clinical safety assessment of another HDACi Vorinostat [54], raises questions about the systemic effects of HDAC inhibition on the tumor microenvironment and immune-cell compartments. We speculate that changes in T cell progenitors in the bone marrow may contribute to the observed phenotype, suggesting a broader impact of HDAC inhibition beyond tumor cells alone. Moreover, the fact that prolonged effects of HDACi were observed months after cessation of the 2-week treatment of young mice, suggests that the treatment eradicated a transient early developmental progenitor cell or even early transformed lymphoma stem cells [55], which would normally give rise to NPM::ALK lymphoma.

Our results challenge the conventional paradigm of HDACs primarily functioning as transcriptional repressors. Deletion of *Hdac1* did not lead to the anticipated stochastic genome-wide chromatin opening but rather resulted in both transcriptional repression and upregulation of gene expression. Our findings support a model wherein HDACs, in conjunction with HATs, play a crucial role in maintaining the delicate balance of histone acetylation patterns, thereby dynamically regulating gene transcription [56–58].

Some of the observed effects might also stem from indirect consequences of HDAC depletion, such as activation of transcriptional activators or loss of repressive factors, which would result in transcriptional activation of target genes. Additionally, HDACs target non-histone proteins and changes in overall protein acetylation might contribute to the observed phenotype. Indeed, in a previous study, we identified several hundred differentially acetylated proteins, including chromatin-modifying proteins and transcription factors, in mouse NPM::ALK tumor cell lines depleted for HDAC1 [19].

The loss of *Hdac1* selectively perturbed T cell-specific transcription, in line with previous studies demonstrating the essential function of HDACs to maintain lineage-specific gene expression in rhabdomyosarcoma [59, 60]. Notably, depletion of HDAC1 protein or loss of its catalytic activity resulted in significant alterations of the immunophenotype of ALK+ tumor cells, including a higher number of TCRb expressing cells and consequently increased dissemination of tumor cells into distant organs. The switch in immunophenotype and the hyperactivated oncogenic signaling including the PDGFRB-STAT5-IL10 oncogenic axis might suggest epigenetic reprogramming of ALK+ tumor cells upon loss of HDAC1. Alternatively, the perturbed T cell development might have resulted in the transformation of a different T cell lineage in *Hdac1*KO thymocytes. The latter would underscore the potential of NPM::ALK to transform a variety of T cell subtypes, which is reflected in controversial findings regarding the cell or origin of ALK+ ALCL [38, 61–64].

In conclusion, our study sheds light on the intricate roles of HDAC1 and HDAC2 as tumor suppressors in ALCL development and highlights the therapeutic potential of HDAC inhibitors, such as Entinostat, in this context. Notably, Entinostat showed similar efficacies in patient-derived cell lines, which were either sensitive or resistant to the ALK inhibitor Crizotinib. Thus, further elucidation of the underlying mechanisms and exploration of combinatorial therapeutic approaches are warranted to optimize treatment strategies for ALCL and other hematological malignancies.

## Supplementary information

Supplementary Methods

Supplementary Figures and Tables

## Data Availability

RNA-seq data: https://www.ncbi.nlm.nih.gov/geo/query/acc.cgi?acc=GSE271907. ATAC-sec data: https://www.ncbi.nlm.nih.gov/geo/query/acc.cgi?acc=GSE271908.
