# Supplementary material for: HDAC1 acts as a tumor suppressor in ALK-positive anaplastic large cell lymphoma: implications for HDAC inhibitor therapy
Source: Leukemia. 2025 Apr 2;39(6):1412–24. doi: 10.1038/s41375-025-02584-9 (PMC12133565; doi:10.1038/s41375-025-02584-9)
Supplement: Supplementary file 1 — Supplementary Methods [file 41375_2025_2584_MOESM1_ESM.pdf]

SUPPLEMENTARY METHODS

Human tissue micro array (TMA) and IHC

The IHC stainings were performed as sequential double-stainings, using HDAC1- and HDAC2- specific antibodies in the first round, CD3- and CD30-specific antibodies in the second round, respectively. TMAs containing ALK+ and ALK- ALCL samples were stained for HDAC1/2 and CD30, while TMAs containing PTCL and AITL samples were stained for HDAC1/2 and CD3 protein expression.

First Round detection: BOND Polymere Refine Detection, Leica Biosystems DS9800

Second Round detection: BOND Polymere Refine Red Detection, Leica Biosystems DS9390

Antibodies used:

CD3 (Leica Biosystems NCL-L-CD3-565), 1:100 heat-induced epitope retrieval (HIER) 20min Epitope Retrieval Sol.1, Leica Biosystems AR9961

CD30 (DAKO M0751), 1:200 HIER 20min Epitope Retrieval Sol.2, Leica Biosystems AR9640

HDAC1 (kind gift of Christian Seiser, clone 10E2), 1:200 HIER 20min Epitope Retrieval Sol.2, Leica Biosystems AR9640

HDAC2 (kind gift of Christian Seiser, clone 3F3), 1:200 HIER 20min Epitope Retrieval Sol.2, Leica Biosystems AR9640

scRNA-seq data analysis

scRNA-seq data generated from $CD45^+$ cells of two primary lymph nodes derived from two patients with ALK+ ALCL**(1)** were retrieved from the Sequence Read Archive (SRA) project PRJNA980417 and analyzed separately. Filtered count matrices were produced using CellRanger v7.1.0 as implemented in the nf-core/scrnaseq pipeline v2.5.1**(2)**. Matrices were processed using Seurat package v5.0.1. After performing QC by removing doublets (doubletFinder package) and cells with low or extremely high RNA features (<500 or >10.000) and with high mitochondrial read fraction (>15), we applied SCTransform normalization, and corrected regressing for cell cycle. Dimensionality reduction was initially performed by Principal Component Analysis (PCA). We then determined the PC in which the percentage change in variation between consecutive PCs is less than 0.1% as the minimum number of PCs that cover most of the variation in the data (nPC =16 for patient 1, nPC =22 for patient 2). Uniform Manifold Approximation and Projection (UMAP) was then applied on the first nPC components of the PCA as determined above. Clusters were identified using the Louvain algorithm with a resolution parameter equal to 2. Cell identity was assessed with SingleR package, using as a reference the Monaco Immune dataset from celldex package, consisting of bulk RNA-seq samples of sorted immune cell populations (GSE107011)**(3)**. ALK+ ALCL cells were defined by stringent filtering by assigning clusters that showed expression of both ALK+ ALCL cell-specific markers ALK and TNFRSF8 (CD30).

Mice genotyping

All mice were genotyped twice, after weaning and after *exitus*. DNA was isolated with DirectPCR Lysis Reagent (Tail) (Viagen, cat#102-T) from ear or tail tissue. Genotyping was performed with GoTaq® Green Master Mix (Promega) according to the manufacturer's suggestions. The PCR products were visualized on 2% agarose gels containing 0.01 μL/mL Midori Green (Nippon Genetics Europe #MG04).

Genotyping was performed with the following primers:

ALK forward: 5'-GGTTCAGGGCCAGTGCATAT-3'

ALK reverse: 5'-CTGGCCTTCATACACCTCCC-3'

CRE forward: 5'-ATGCTTCTGTCCGTTTGCCG-3'

CRE reverse: 5'-TGAGTGAACGAACCTGGTCG-3'

Hdac1 forward: 5'-GGTAGTTCACAGCATAGTACTT-3'

Hdac1 reverse: 5'-CCTGTGTCATTAGAATCTACTT-3'

Hdac2 forward: 5'-GGTAGTTCACAGCATAGTACTT-3'

Hdac2 reverse: 5'-GTTACGTCAATGACATCGTCTT-3'

Hdac1 KI screen forward: 5'-GCATCGCCTTCTATCGCCTTC-3'

Hdac1 KI screen reverse: 5'-CTTGGTCATCTCCTCAGCATTGG-3'

Rosa26 screen forward: 5'-AAGAACTGCAGTGTTGAGGC-3'

Rosa26 screen reverse: 5'-TCTCCCAAAGTCGCTCTGAG-3'

Establishing and treatment of murine NPM-ALK cell lines

ALK cell lines were established from NPM-ALK *Hdac1*fl/fl murine T cell lymphomas and transduced with tamoxifen-inducible Cre as previously described(4). ALK cell lines

were treated with the HDACi Vorinostat (Selleckchem), Valproic Acid (Sigma), Entinostat (Selleckchem) and Romidepsin (Selleckchem) with previously determined IC50s for 16h before harvest.

Immunohistochemistry (IHC)

After fixation in 4.5 % neutral buffered formaldehyde solution tissues were embedded in paraffin and cut into 5 µM sections. Following deparaffinization and rehydration, antigen retrieval was performed by heat-treatment in TRIS/EDTA (Target Retrieval Solution pH 9 Dako) or citrate buffer (Target Retrieval Solution pH 6 Dako). Endogenous peroxidase was blocked by 3% H_2O_2, slides were blocked with avidin/biotin (Vector lab), super block (Empire Genomics) and universal mouse block (Empire Genomics). Slides were incubated with primary antibody o/n at 4°C, followed by secondary antibody and incubation with the IDetect™ Super Stain System - HRP kit (Empire Genomics). The staining was performed with AEC reagent (Empire Genomics), followed by counterstain by incubation in Mayer's hemalum solution (Merck).

Antibodies used:

Ki67 (D3B5) Cell Signaling #9129 1:400 dilution

CC3 (ASP175) Cell Signaling #9661 1:200 dilution

ALK (D5F3) Cell Signaling #3633 1:250 dilution

Protein isolation, Western blotting – buffers composition and antibodies

Buffer compositions:

Hunt buffer: 20 mM Tris pH 8, 100 mM NaCl, 1 mM EDTA, 0.5% NP-40, protease inhibitor - Roche

Milk blocking solution: 5% skim milk powder, 1% PVP, 0.01% Sodium azide in TBS-T (=TBS containing 1% Triton-X100)

BSA blocking solution: 5% BSA, 0.01% Sodium azide in TBS-T (=TBS containing 1% Triton-X100)

*BSA blocking solution was used when phosphorylated proteins were detected.

ECL solutions: Amersham ECL Detection Reagents or Bio-Rad Clarity ECL substrate (to detect pSTAT5)

Antibodies used:

HDAC1 (kind gift of Christian Seiser, clone 10E2), dilution 1:1000

HDAC2 (kind gift of Christian Seiser, clone 3F3) dilution 1:1000

ALK Cell Signaling #4691, dilution 1:2500

p-ALK Cell Signaling #6941, dilution 1:500

STAT3 Cell Signaling #12640, dilution 1:1000

p-STAT3 Cell Signaling #9131, dilution 1:1000

CD3g Proteintech #21120-AP, dilution 1:1000

CD3d Proteintech #16669-I-AP, dilution 1:1000

CD3e (SP7) Abcam #ab16669, dilution 1:1000

PDGFRb Cell Signaling #3169, dilution 1:1000

STAT5a (C-6) Santa Cruz #sc-271542, dilution 1:10000

STAT5b (G-2) Santa Cruz #sc-1656, dilution 1:1000

pSTAT5 Cell Signaling #9351, dilution 1:1000

NFAT1 Cell Signaling #4389, dilution 1:1000

c-MYC (E5Q6W9) Cell Signaling #18583, dilution 1:500

Beta Actin (Proteintech #66009-I-Ig), dilution 1:5000

Alpha Tubulin (Proteintech #66031-1-Ig), dilution 1:5000

Goat anti-rabbit horseradish peroxidase-linked secondary antibody (Jackson Immuno Research, #111-036-047), dilution 1:10,000

Rabbit anti-mouse horseradish peroxidase-linked secondary antibody (Jackson Immuno Research, #315-035-045), dilution 1:10,000

Histone isolation, Western blotting – buffers composition and antibodies

Buffer compositions:

Lysis buffer: 10 mM Tris pH 6.5, 50 mM Na_2S2O_5, 10 mM $MgCl_2$, 1% Triton X-100, 8.6% sucrose, adjusted to pH 6.5; right before use the following inhibitors were added: 2 mM Na_3VO_4, 10 mM NaF, 0.2 mM PMSF, 1x Protease inhibitor - Roche, 5 mM Na-Butyrate

Wash buffer: 10 mM Tris pH 7.4, 13 mM Na_3EDTA, adjusted to pH 7.4

Antibodies used:

H3ac (pan) Active Motif #61637, dilution 1:1000

H4ac (pan) Active Motif #39026, dilution 1:1000

H2B Cell Signaling #12364, dilution 1:1000

Goat anti-rabbit horseradish peroxidase-linked secondary antibody (Jackson Immuno Research, #111-036-047), dilution 1:10,000

Rabbit anti-mouse horseradish peroxidase-linked secondary antibody (Jackson Immuno Research, #315-035-045), dilution 1:10,000

ATAC-seq and RNA-seq sample

Snap frozen tumor tissue was lyzed in homogenization buffer. Nuclei were pelleted by centrifugation (5 min, 4°C, 350 rcf). The supernatant was used to isolate RNA (see below). Nuclei were resuspended in homogenization buffer and gradients of iodixanol solution were used to obtain the band containing the nuclei at the 30 – 40% iodixanol interface after centrifugation (20 min, 4°C, 3000 rcf, break off). Nuclei were diluted in ATAC-RSB-Tween buffer and counted. 50,000 nuclei per sample were centrifuged (10 min, 500 rcf, 4°C) and resuspended in ATAC-seq Reaction Mix. Reactions were incubated (37°C, 30 min, 1000 rpm). After incubation, Binding Buffer (Qiagen MinElute PCR Purification kit) was added. A cleaning protocol using the MiniElute PCR Purification kit was performed according to the manufacturer and samples were eluted in Elution Buffer.

RNA isolation: 150 μL of the supernatant (see above) was mixed with Trizol and chloroform, vortexed and centrifuged (15 min, 4°C, 21,000 rcf). The aqueous layer was mixed with an equal volume of 100% ethanol and passed through a QIAgen RNeasy column. The QIAgen RNeasy protocol was followed and samples were eluted in 27 μL Elution Buffer. 3 μL of 10 x Turbo™ DNase Buffer and 1 μL of Turbo™ Dnase enzyme was added and the samples were incubated (30 min, 37°C). 70 μL of RNase-free water and 350 μL of QIAgen RLT Buffer were added. 250 μL of 100% ethanol

was added and the samples were applied to the column and washed 2 x with RPE. Samples were eluted in RNase-free water.

Buffer compositions:

Homogenization buffer: final concentration 0.26 M sucrose, 0.03 M KCl, 0.01 M $MgCl_2$, 0.02 M Tricine-KOH pH 7.8, with 0.001 M DTT, 0.5 mM spermidine, 0.15 mM spermine, 0.3 % NP40, 1x cOmplete™ Protease Inhibitor, 10 μL RiboLock per mL of buffer

Iodixanol solution: iodixanol diluted with appropriate amount of diluent buffer

Diluent buffer: 0.15 M KCl, 0.03 M $MgCl_2$, 0.12 M Tricine-KOH pH 7.8

ATAC-RSB-Tween buffer: final concentration 0.01 M Tris-HCl pH 7.5, 0.01 M NaCl, 0.003 M $MgCl_2$, 0.1 % Tween-20 in water

ATAC-seq Reaction Mix per sample: 5 μL water, 16.5 μL PBS, 25 μL 2x TD, 0.5 μL 1% digitonin, 0.5 μL 10% Tween-20, 2.5 μL Tn5

RNA-seq data analysis

Cutadapt(5) was used to remove unwanted sequences (e.g., adapters, poly-A tails, etc.) and low-quality reads on the FASTQ files provided by the sequencing facility. FASTQC(6) and MULTIQC(7) were both used to check the quality of reads. The STAR aligner tool(8) was used to map the reads to the GRCm39 mouse reference genome, obtained from ENSEMBL (https://www.ensembl.org/Mus_musculus/Info/Index). Htseq-count(9) was used to obtain gene counts. Finally, we performed a naive pre-filtering step to remove low count genes by only keeping those transcripts with an average count across all samples bigger than one. We used the R/Bioconductor

package DESeq2(10) to perform differential gene expression (DE) analysis of the Htseq transcript counts. Genes with an adjusted P-value (p adj) < 0.05 and absolute log2 fold change (logFC)≥1 were considered significantly differentially expressed. Finally, read counts were normalized by the DESeq2 normalization method of variance stabilizing transformation (VST), to be used primarily for visualization. Figures for this analysis were generated using DESeq2.

ATAC-seq data analysis

Initial data quality check was done using FastQC(6). Next, transposase (Tn5) sequences were removed using the cutadapt tool(5). All Illumina adapters were likewise removed. Next Bowite2(11) was used for mapping reads to the reference genome. Sorting of the mapped reads was done using samtools(12). Mitochondrial reads and sex chromosomes as well as reads with low mapping quality (MAPQ < 30) were removed. For deduplication rmdup and samtools(12,13) were used. Peaks were called with MACS2 (Model-based analysis of ChIP-seq)(14). FRiP scores were calculated and quality check was performed using multiQC(7). Counting reads per peak was accomplished with featureCounts(15). To annotate peaks, the package ChIPseeker(16) was used. Differential enrichment analysis was done using edgeR(17). Motif analysis was done using HOMER software(18).

Correlation of RNA-seq and ATAC-seq data

Correlation was done using the software developed by Okonechnikov et al.(19). Publicly available *Mus musculus* HiC data from the NCBI GEO Database (accession no. GSE105918)(20) were used. Both RNA- and ATAC-seq were normalized using

the TPM+log2 method(21). The median of all transcript lengths per particular gene was used to calculate transcript length per gene. Consensus peak regions were inferred from ATAC-seq data using DiffBind (v.3.0)(22). Correlations with $p < 0.05$ were considered significant.

Functional gene set enrichment analysis

Ingenuity Pathway Analysis (IPA®,Qiagen) was performed including deregulated genes (RNA-seq: p adj < 0.05 and logFC $\geq$ I1I), which showed a correlation with changes in chromatin accessibility (p for correlation < 0.05).

HDAC activity assay

20 µg/20 µL of protein were incubated with 4 µL of ^{3}H-acetate–labelled chicken erythrocyte histone mix (1.5 mg/mL) for 1 h at 30°C on a thermoshaker at 300 rpm. The reaction was stopped by adding 35 µL of histone stop solution and 800 µL ethyl acetate. The samples were vortexed and centrifuged (4 min, 10000 rpm). 600 µL of the organic phase was transferred to 3 mL of scintillation solution and mixed gently. The HDAC activity was determined by a Liquid Scintillation Analyzer.

FACS Immunophenotyping – preprocessing of the samples

Single cell suspensions of thymus, tumor and spleen were obtained by passaging the tissues through a 70 µm nylon cell strainer in staining buffer (PBS supplemented with 2% FCS). Bone marrow was isolated using empty DMEM medium following the STAR protocol(23). Erythrocytes were lysed (5 min on ice) using erylysis buffer (final

concentrations 0.15 M NH_4Cl, 10 mM $KHCO_3$, 0.1 mM EDTA, pH = 7.2 – 7.4) before staining. Cells were stained for viability and cell surface markers (20 min, RT). Cells were fixed with FoxP3 Transcription Factor Fixation/Permeabilization Solution (eBioscience) and stained for ALK (30 min, 4°C), followed by Alexa Fluor 647 goat anti-mouse IgG antibody (30 min, 4°C).

FACS Immunophenotyping – antibodies

Antibodies used:

ANTIGEN	FLUOROPHORE	CLONE	COMPANY
viability	Zombie NIR	/	BioLegend
CD45	BV650	30-F11	BD Biosciences
CD19	APC-Cy7	6D5	BioLegend
CD11b	PerCP-Cy5.5	M1/70	Invitrogen Antibodies
Gr-1	PE	RB6-8C5	Invitrogen Antibodies
CD117 = cKit	PECY5	2B8	Invitrogen Antibodies
Sca1	PE-Cy7	D7	Invitrogen Antibodies
CD34	AF700	RAM34	Invitrogen Antibodies
CD16/32	PerCP-eF710	93	Invitrogen Antibodies
CD127 = IL7R	FITC	A7R34	Invitrogen Antibodies
TCRb	BV510	H57-597	BD Biosciences
TCRgd	BV711	GL3	BD Biosciences
CD8a	BUV496	53-6.7	BD Biosciences
CD4	BUV395	GK1.5	BD Biosciences
CD25	PB	PC61	BioLegend
CD44	BV570	IM7	BioLegend
ALK (cell signaling#3633) + AF647 goat anti-rabbit (Invitrogen#a-21244)	AF647	/	Invitrogen
CD69	BV785	H1.2F3	BioLegend
CD62L	BV605	MEL-14	BD Biosciences

FACS Immunophenotyping – tSNE

For unsupervised clustering, first the thymic tumor samples were downsized using FlowJo™ (BD Life Sciences) plugin "Downsample" (version 3.3.1). 100.000 of events were used from the gating for alive leukocytes (CD45+ cells, negative for viability stain). New Downsample populations were concatenated (using all events and all compensated parameters). Next tSNE analysis (part of FlowJo™, BD Life Sciences) was performed on concatenated sample. To run tSNE, all markers were used except viability and CD45.

***In vivo* HDACi treatments**

Mice were treated with HDACi for two consecutive weeks on a five-days-on-two-days-off schedule. Entinostat (Selleckchem) was administered via interperitoneal (IP) injection, with a 1 mL single use Tuberculin syringe (Chirana) and a 27-gauge (G) x ¾" (0.4 mm x 20 mm) safety needle (SOL-CARE). Entinostat (Selleckchem) was aliquoted beforehand and diluted daily prior to injection. The drug was diluted in 90% sterile filtered corn oil (Mazola) and 10% DMSO. Dilutions for different treatment doses can be seen in Table 1. The volumes injected based on mouse weight are shown in Table 2 (example for 10 μg/g treatment dose).

Table 1

Treatment group	mg of substance/mL DMSO+oil (10/90)
Entinostat 50 μg/g	10 mg/mL
Entinostat 20 μg/g	4 mg/mL
Entinostat 10 μg/g	2 mg/mL
Entinostat 5 μg/g	1 mg/mL

Table 2

Body weight (g)	Dose (μg)	Vol. injection (μL)
15	150	75
16	160	80
17	170	85
18	180	90
19	190	95
20	200	100
21	210	105
22	220	110
23	230	115
24	240	120
25	250	125

Electron Microscopy

After fixation in Karnovsky solution, tissues were postfixed in 1% osmium ferrohexacyanoferrate II, then washed in distillated water and dehydrated in ethanol and propylene oxide and embedded in Epon resin (Embed 812). Ultrathin sections of 70 nm were made on a Leica-Ultracut- EM-UC7. For contrast enhancement, the ultrathin sections were stained in 2% uranyl acetate and 1% lead citrate. Transmission electron microscopy was then performed on a TEM Jeol 1400 Plus (Jeol) at 60kV, pictures were taken with Quemesa_Camera in radius-software.

Statistics

Survival statistics were analyzed using GraphPad Prism (version 8.4.3). To assess differences between groups pairwise curve comparison using Log-rank (Mantel–Cox) test was performed. Data are represented as mean ± SD, if not otherwise specified and were analyzed using GraphPad Prism (version 8.4.3). To assess differences between groups, unpaired t test or one-way ANOVA were used, depending on number

of sample groups. Significance was defined according to following P-values: $*P < 0.05$; $**P < 0.01$; $***P < 0.001$ $****P < 0.0001$.

REFERENCES

1. Mastini C, Campisi M, Patrucco E, Mura G, Ferreira A, Costa C, et al. Targeting CCR7-PI3Kγ overcomes resistance to tyrosine kinase inhibitors in ALK-rearranged lymphoma. Sci Transl Med. 2023 Jun 28;15(702):eabo3826.

2. Ewels PA, Peltzer A, Fillinger S, Patel H, Alneberg J, Wilm A, et al. The nf-core framework for community-curated bioinformatics pipelines. Nat Biotechnol. 2020 Mar;38(3):276–8.

3. Monaco G, Lee B, Xu W, Mustafah S, Hwang YY, Carré C, et al. RNA-Seq Signatures Normalized by mRNA Abundance Allow Absolute Deconvolution of Human Immune Cell Types. Cell Rep. 2019 Feb 5;26(6):1627-1640.e7.

4. Zrimšek M, Kuchaříková H, Draganić K, Dobrovolná P, Heiss Spornberger V, Winkelmayer L, et al. Quantitative Acetylomics Uncover Acetylation-Mediated Pathway Changes Following Histone Deacetylase Inhibition in Anaplastic Large Cell Lymphoma. Cells. 2022 Jan;11(15):2380.

5. Martin M. Cutadapt removes adapter sequences from high-throughput sequencing reads. EMBnet.journal. 2011 May 2;17(1):10–2.

6. Andrews, S. (2010). FastQC: A Quality Control Tool for High Throughput Sequence Data [Online]. Available online at: http://www.bioinformatics.babraham.ac.uk/projects/fastqc/.

7. Ewels P, Magnusson M, Lundin S, Käller M. MultiQC: summarize analysis results for multiple tools and samples in a single report. Bioinformatics. 2016 Oct 1;32(19):3047–8.

8. Dobin A, Davis CA, Schlesinger F, Drenkow J, Zaleski C, Jha S, et al. STAR: ultrafast universal RNA-seq aligner. Bioinformatics. 2013 Jan;29(1):15–21.

9. Anders S, Pyl PT, Huber W. HTSeq—a Python framework to work with high-throughput sequencing data. Bioinformatics. 2015 Jan 15;31(2):166–9.

10. Love MI, Huber W, Anders S. Moderated estimation of fold change and dispersion for RNA-seq data with DESeq2. Genome Biol. 2014 Dec 5;15(12):550.

11. Langmead B, Salzberg SL. Fast gapped-read alignment with Bowtie 2. Nat Methods. 2012 Apr;9(4):357–9.

12. Danecek P, Bonfield JK, Liddle J, Marshall J, Ohan V, Pollard MO, et al. Twelve years of SAMtools and BCFtools. GigaScience. 2021 Feb 1;10(2):giab008.

13. Li H, Handsaker B, Wysoker A, Fennell T, Ruan J, Homer N, et al. The Sequence Alignment/Map format and SAMtools. Bioinformatics. 2009 Aug 15;25(16):2078–9.

14. Gaspar JM. Improved peak-calling with MACS2 [Internet]. bioRxiv; 2018 [cited 2024 Feb 21]. p. 496521. Available from: https://www.biorxiv.org/content/10.1101/496521v1

15. Liao Y, Smyth GK, Shi W. featureCounts: an efficient general purpose program for assigning sequence reads to genomic features. Bioinformatics. 2014 Apr 1;30(7):923–30.

16. Yu G, Wang LG, He QY. ChIPseeker: an R/Bioconductor package for ChIP peak annotation, comparison and visualization. Bioinformatics. 2015 Jul 1;31(14):2382–3.

17. Robinson MD, McCarthy DJ, Smyth GK. edgeR: a Bioconductor package for differential expression analysis of digital gene expression data. Bioinformatics. 2010 Jan 1;26(1):139–40.

18. Heinz S, Benner C, Spann N, Bertolino E, Lin YC, Laslo P, et al. Simple Combinations of Lineage-Determining Transcription Factors Prime cis-Regulatory Elements Required for Macrophage and B Cell Identities. Mol Cell. 2010 May 28;38(4):576–89.

19. Okonechnikov K, Erkek S, Korbel JO, Pfister SM, Chavez L. InTAD: chromosome conformation guided analysis of enhancer target genes. BMC Bioinformatics. 2019 Jan 31;20(1):60.

20. Johanson TM, Coughlan HD, Lun ATL, Bediaga NG, Naselli G, Garnham AL, et al. Genome-wide analysis reveals no evidence of trans chromosomal regulation of mammalian immune development. PLOS Genet. 2018 Jun 8;14(6):e1007431.

21. Wagner GP, Kin K, Lynch VJ. Measurement of mRNA abundance using RNA-seq data: RPKM measure is inconsistent among samples. Theory Biosci. 2012 Dec 1;131(4):281–5.

22. Ross-Innes CS, Stark R, Teschendorff AE, Holmes KA, Ali HR, Dunning MJ, et al. Differential oestrogen receptor binding is associated with clinical outcome in breast cancer. Nature. 2012 Jan;481(7381):389–93.

23. Toda G, Yamauchi T, Kadowaki T, Ueki K. Preparation and culture of bone marrow-derived macrophages from mice for functional analysis. STAR Protoc. 2021 Mar 19;2(1):100246.
