# Supplementary material for: HDAC1 acts as a tumor suppressor in ALK-positive anaplastic large cell lymphoma: implications for HDAC inhibitor therapy
Source: Leukemia. 2025 Apr 2;39(6):1412–24. doi: 10.1038/s41375-025-02584-9 (PMC12133565; doi:10.1038/s41375-025-02584-9)
Supplement: Supplementary file 2 — Supplementary Figures and Tables [file 41375_2025_2584_MOESM2_ESM.pdf]

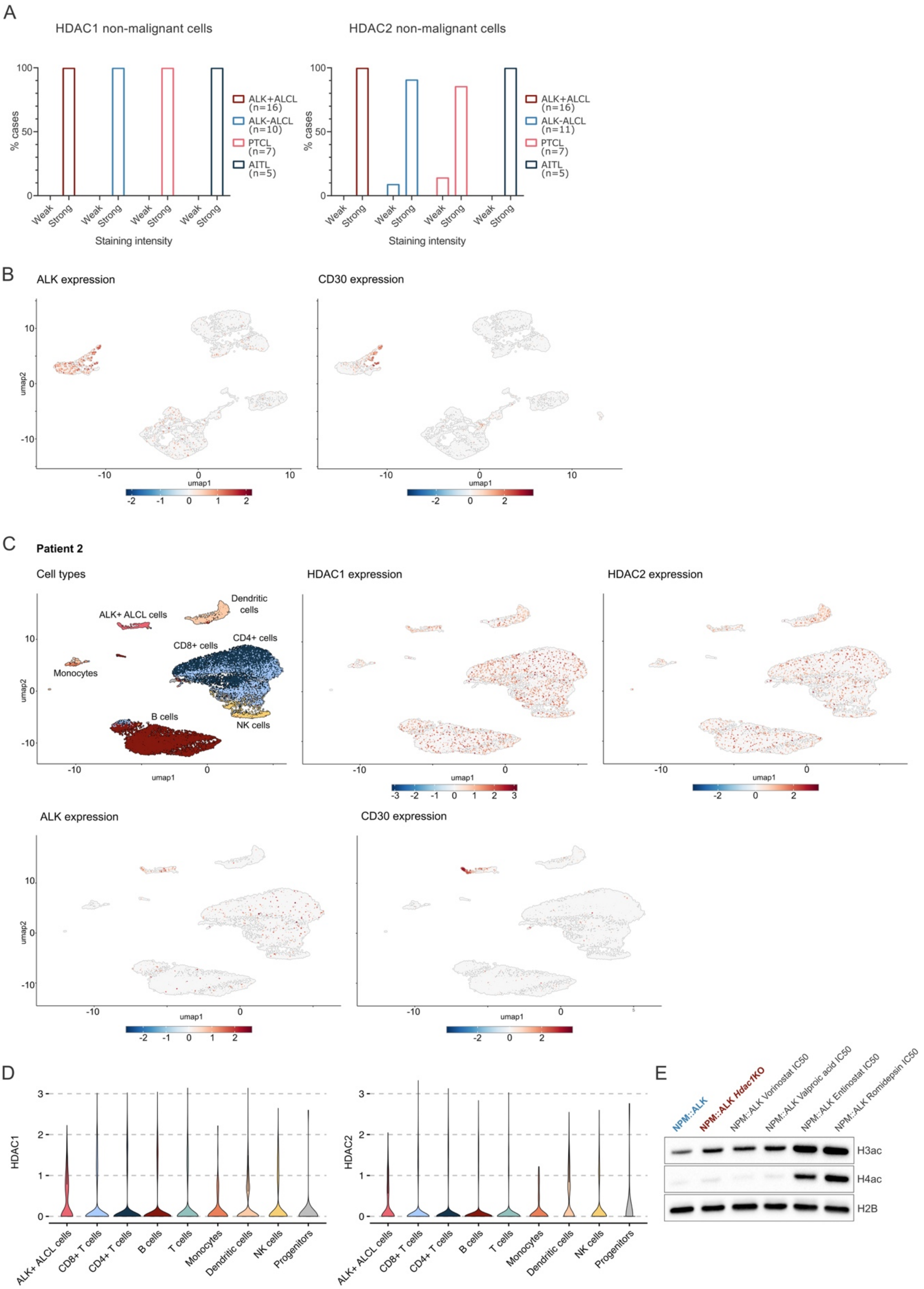

SUPPLEMENTAL FIGURE S1: HDAC inhibitor treatment before tumor onset significantly restricts NPM::ALK tumor development

(A) Bar plots depicting the percentages of HDAC1 (left) or HDAC2 (right) staining intensities (weak, strong) of non-malignant cells on tissue microarrays (TMAs) containing specified

numbers of ALK+ ALCL, ALK- ALCL, PTCL, and AITL patient samples, evaluated by immunohistochemistry (IHC) (as in main Figure 1A). Non-malignant cells were determined by lack of CD30 staining for ALK+ ALCL, or based on their morphology by a trained hemato-pathologist **(B)** UMAP plots showing levels of normalized expression of ALK (left) and CD30 (right) as seen in scRNA-seq data for $CD45^+$ cells from a primary lymph node of ALK+ ALCL patient #1 (cell types as seen in main Figure 1B). **(C)** (upper) UMAP plots of scRNA-seq data from $CD45^+$ cells from a primary lymph node of ALK+ ALCL patient #2. Left plot shows cells in a dimensional reduction embedding, color-coded according to the different annotated cell types, middle and right plots show levels of normalized gene expression for *HDAC1* and *HDAC2*. (lower) UMAP plots showing levels of normalized expression of ALK (left) and CD30 (right) as seen in scRNA-seq data for $CD45^+$ cells from a primary lymph node of ALK+ ALCL patient #2 (cell types as seen above). **(D)** Violin plots depicting normalized expression of *HDAC1* (left) and *HDAC2* (right) in color-coded cell types as in (B). **(E)** Immunoblot of protein levels of acetylated histone 3 (H3ac) and acetylated histone 4 (H4ac) in a primary cell line isolated from an end-stage thymic tumor of a NPM::ALK mouse. The NPM::ALK cell line was either transformed with inducible CRE and treated with tamoxifen to produce a NPM::ALK *Hdac1*KO cell line or it was treated with IC50 concentrations of HDAC inhibitors including Vorinostat, Valproic Acid, Entinostat and Romidepsin (n=1 for each condition). Histone 2B (H2B) was used as a loading control.

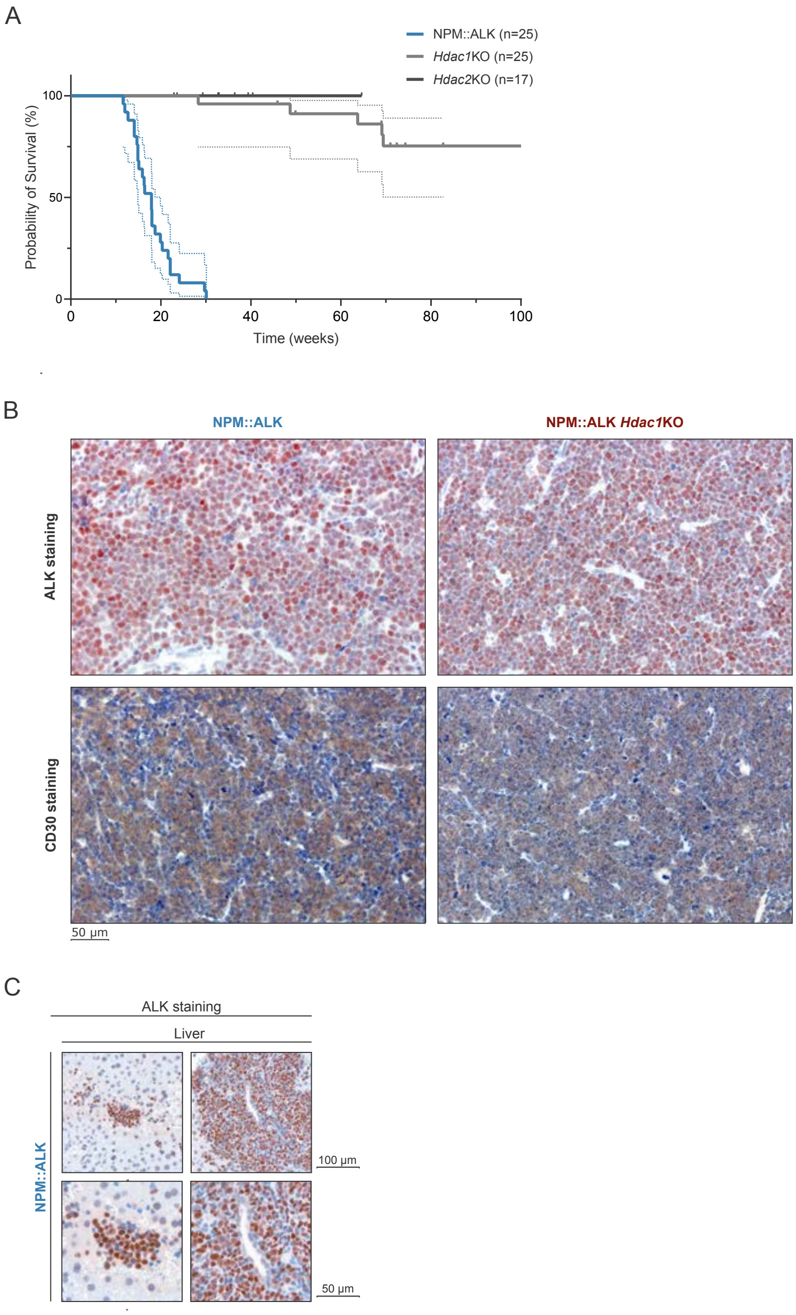

SUPPLEMENTAL FIGURE S2: *Hdac1* loss in T cells accelerates lymphomagenesis

(A) Kaplan Meier survival analysis of NPM::ALK mice (n=25, light blue line), *Hdac1*KO mice (n=25, light gray line) and *Hdac2*KO mice (n=17, dark gray line) in biological replicates. GraphPad Prism version 8.4.3 was used for analysis. (B) Representative microscopic images of end-stage tumor sections from NPM::ALK (n=3) and NPM::ALK Hdac1KO mice (n=3) stained for ALK and CD30 expression by IHC. Sections were counterstained with hematoxylin (blue). Scale bar represents 50 µm. (C) Representative microscopic images of liver sections from NPM::ALK mice (n=2) stained for ALK expression by IHC. Sections were counterstained with hematoxylin (blue). Infiltration of ALK positive cells around vessels is displayed at two magnifications. Scale bar represents 100 µm (upper panel), and 50 µm (lower panel).

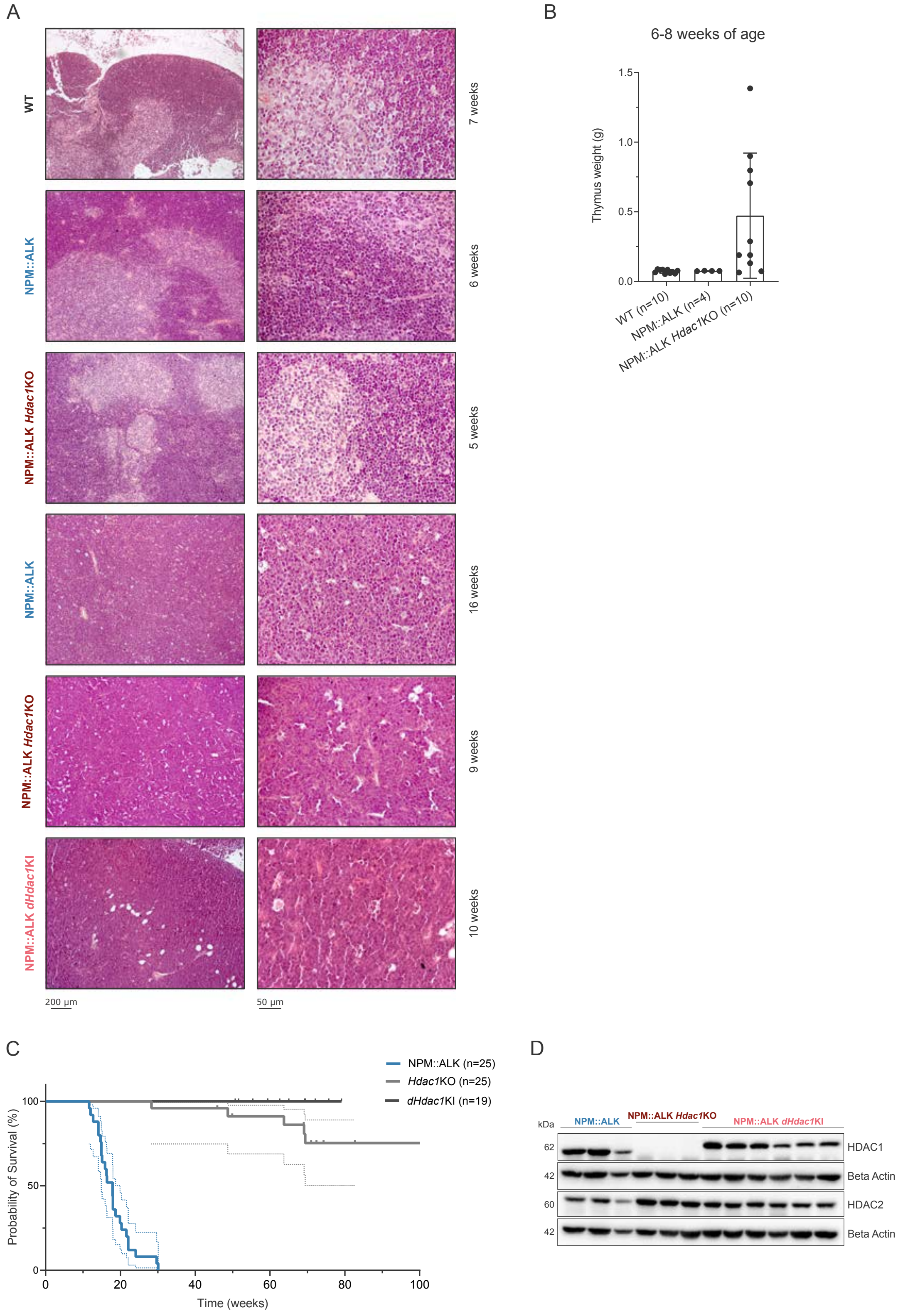

SUPPLEMENTAL FIGURE S3: Accelerated lymphomagenesis depends on HDAC1 enzymatic activity

(**A**) Representative microscopic images of H&E stained sections, displaying thymus architecture of 7-week old WT, 6-week old NPM::ALK, and 5-week old NPM::ALK *Hdac1*KO

mice, compared to end-stage tumors with disrupted thymus architecture of 16-week old NPM::ALK, 9-week old NPM::ALK *Hdac1*KO and 10-week old NPM::ALK *Hdac1*KI mice. (n=3 for each genotype). Scale bars represent 200 μm and 50 μm as indicated at the bottom. (**B**) Comparison of thymic mass (g) of different genotypes of mice between 6 and 8 weeks of age (WT n=10, NPM::ALK n=4, NPM::ALK *Hdac1*KO n=10). Mean with standard deviation (SD) is plotted. (**C**) Kaplan Meier survival analysis of NPM::ALK mice (n=25, blue line), *Hdac1*KO mice (n=25, light gray line) *dHdac1*KI mice (n=19, dark gray line) in biological replicates. GraphPad Prism version 8.4.3 was used for analysis. (**D**) Immunoblot of protein levels of HDAC1 and HDAC2 in end-stage thymic tumors excised from NPM::ALK (n=4), NPM::ALK *Hdac1*KO (n=4) and NPM::ALK *Hdac1*KI (n=4) mice. Beta-Actin was used as a loading control. The molecular weight of analyzed proteins in kiloDaltons (kDa) is indicated by the numbers on the left. Corresponding quantification of the blots is shown in main Figure 3E.

A

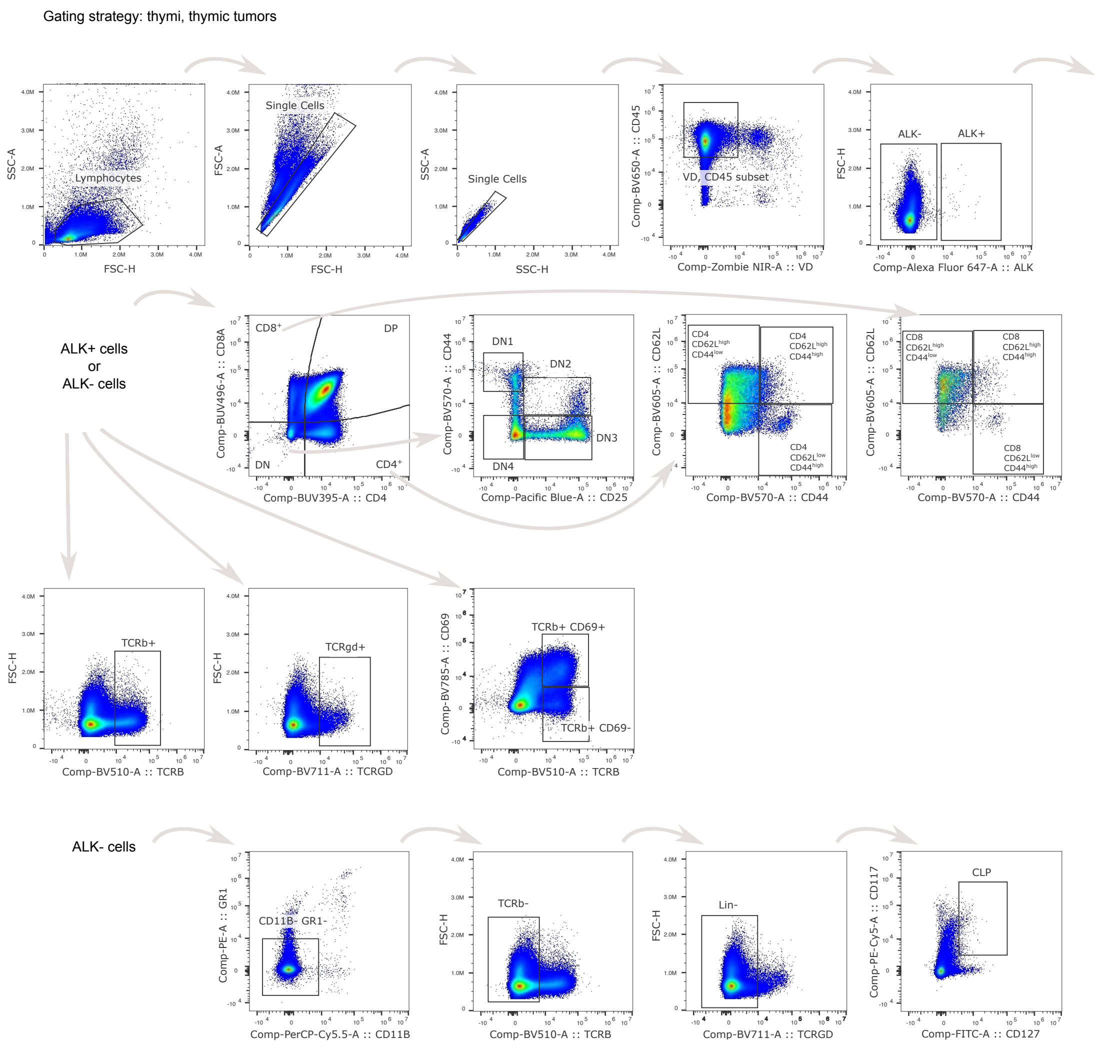
Gating strategy: thymi, thymic tumors
Lymphocytes
SSC-A
FSC-H
Single Cells
FSC-A
FSC-H
Single Cells
SSC-A
SSC-H
VD, CD45 subset
Comp-BV650-A :: CD45
Comp-Zombie NIR-A :: VD
ALK-
ALK+
FSC-H
Comp-Alexa Fluor 647-A :: ALK
ALK+ cells
or
ALK- cells
CD8+
DP
DN
CD4+
Comp-BUV496-A :: CD8A
Comp-BUV395-A :: CD4
DN1
DN2
DN3
DN4
Comp-BV570-A :: CD44
Comp-Pacific Blue-A :: CD25
CD4 CD62Lhigh CD44low
CD4 CD62Lhigh CD44high
CD4 CD62Llow CD44high
Comp-BV605-A :: CD62L
Comp-BV570-A :: CD44
CD8 CD62Lhigh CD44low
CD8 CD62Lhigh CD44high
CD8 CD62Llow CD44high
Comp-BV605-A :: CD62L
Comp-BV570-A :: CD44
TCRb+
FSC-H
Comp-BV510-A :: TCRB
TCRgd+
FSC-H
Comp-BV711-A :: TCRGD
TCRb+ CD69+
TCRb+ CD69-
Comp-BV785-A :: CD69
Comp-BV510-A :: TCRB
ALK- cells
CD11B- GR1-
Comp-PE-A :: GR1
Comp-PerCP-Cy5.5-A :: CD11B
TCRb-
FSC-H
Comp-BV510-A :: TCRB
Lin-
FSC-H
Comp-BV711-A :: TCRGD
CLP
Comp-PE-Cy5-A :: CD117
Comp-FITC-A :: CD127

B

Gating strategy: spleen

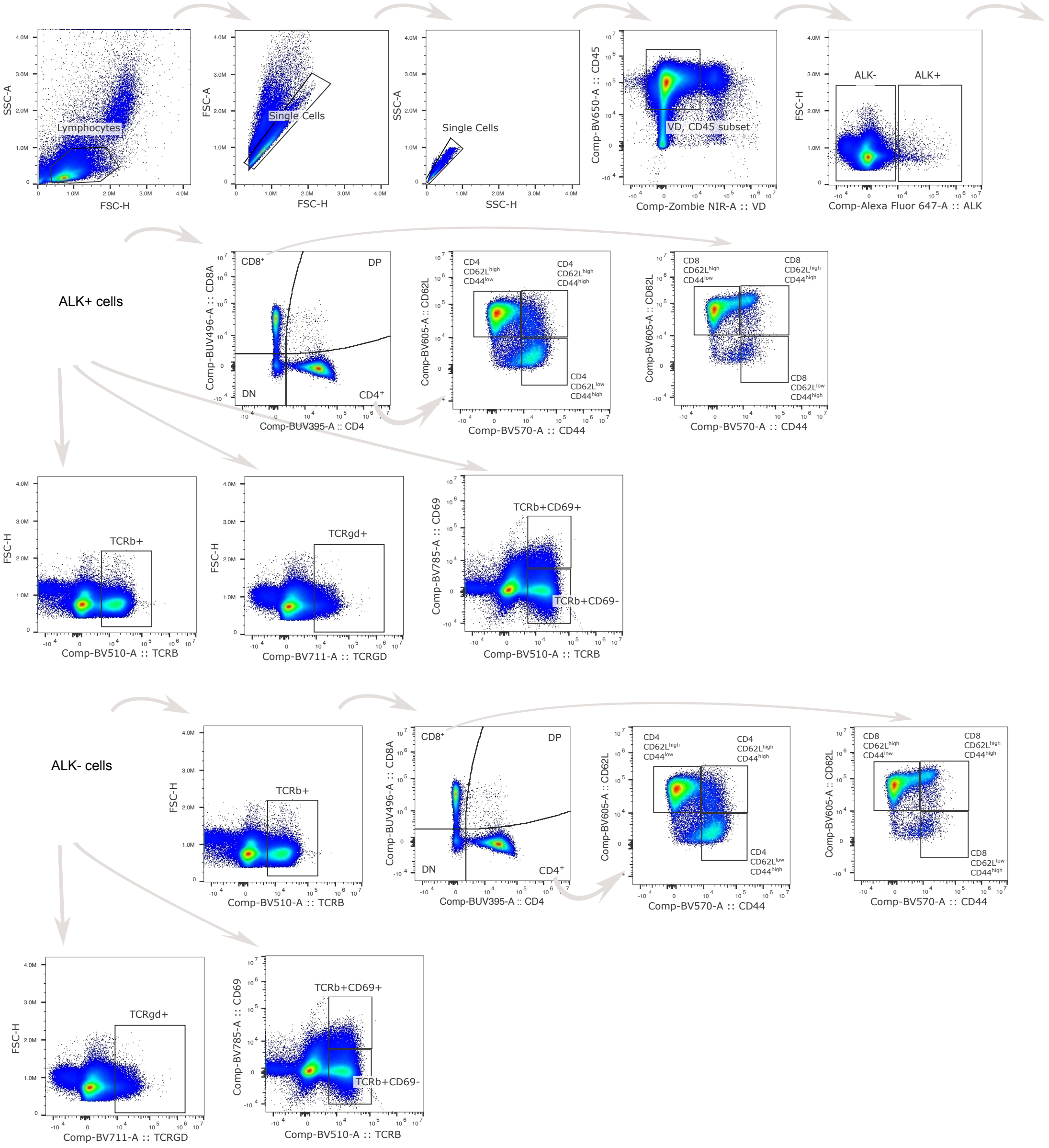

C

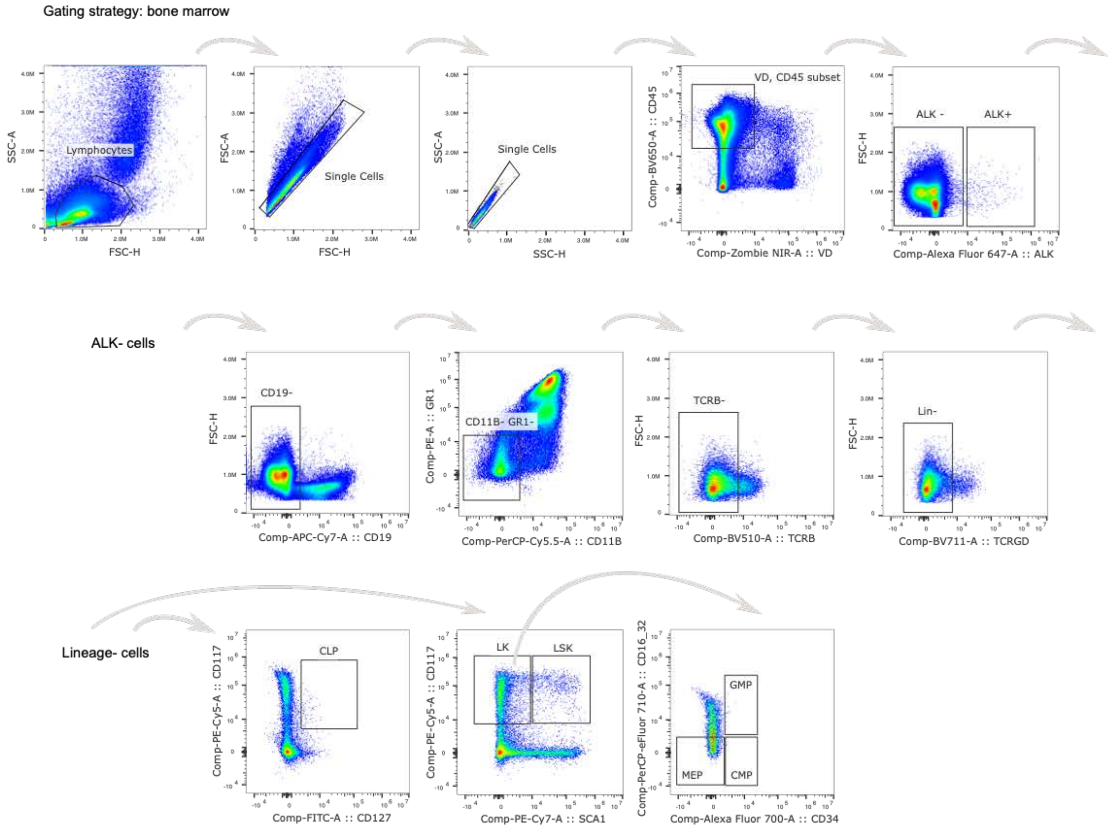

D

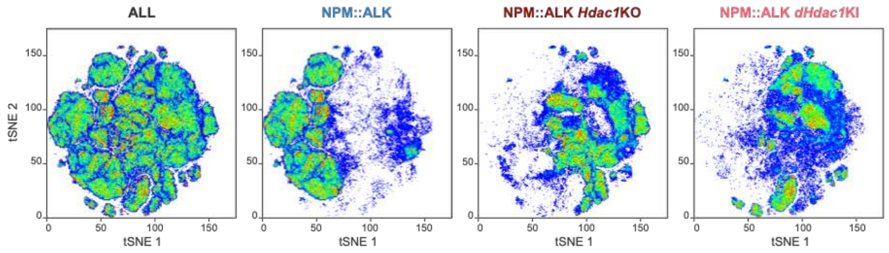

E

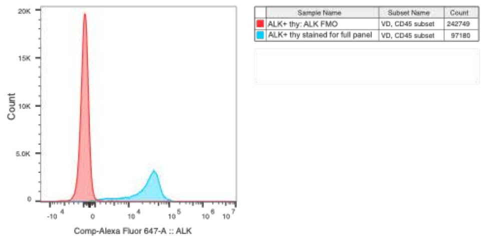

F

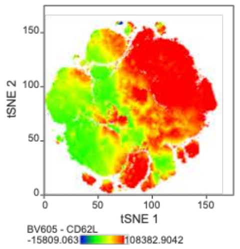

SUPPLEMENTAL FIGURE S4: Loss of HDAC1 protein or HDAC1 catalytic activity causes changes in the immunophenotype

(A) Exemplary gates utilized for analyzing thymic tumor samples, established using WT thymus samples stained and acquired concurrently with tumor samples. **(B)** Exemplary gates employed for analyzing spleen samples, established using WT spleen samples stained and acquired concurrently with samples from tumor-bearing mice. **(C)** Exemplary gates applied for analyzing bone marrow samples, established using WT bone marrow samples stained and acquired concurrently with samples from tumor-bearing mice. **(D)** Unsupervised clustering of

flow cytometry (FACS) immunophenotyping using tSNE plots (live, CD45+ leukocytes from thymic tumors were used for clustering). From left to right, clustering of all samples (n=15) is shown, followed by NPM::ALK only (n=6), NPM::ALK *Hdac1*KO only (n=5) and NPM::ALK *dHdac1*KI only samples (n=4). **(E)** Representative flow cytometry histograms showing ALK expression on live CD45+ NPM::ALK positive thymocytes, stained with either the full panel (blue) or the full panel minus ALK antibody (red), serving as the fluorescence minus one control (FMO control). **(F)** tSNE plot based on CD62L expression (live, CD45+ leukocytes) of all samples (n=15). The color depicts the expression of CD62L with red depicting high expression and green is depicting low expression. Samples as in (D).

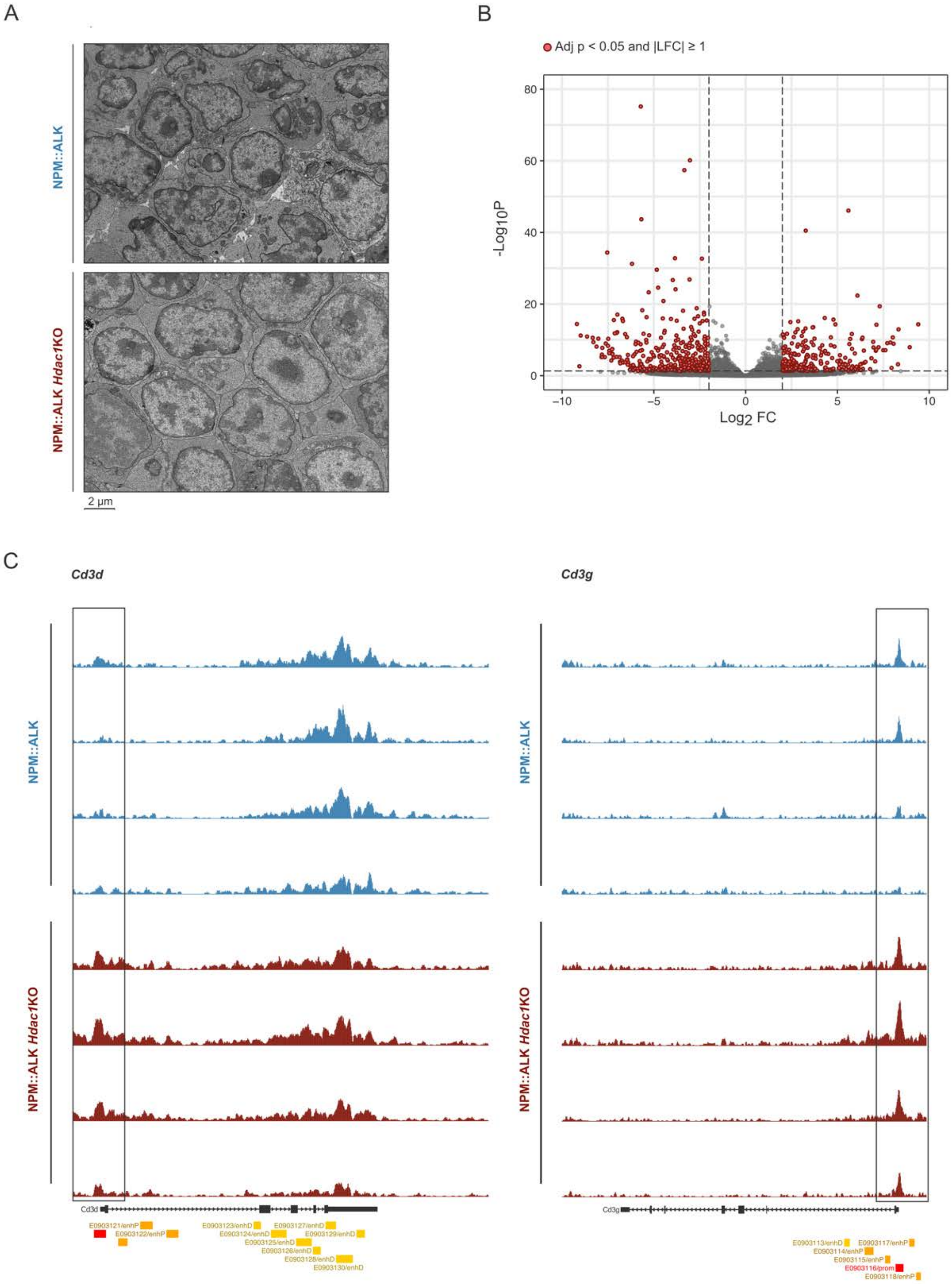

SUPPLEMENTAL FIGURE S5: Loss of *Hdac1* selectively perturbs cell-type specific transcription (A) Representative transmission electron microscopy pictures of NPM::ALK (n=1) and NPM::ALK *Hdac1*KO (n=1) end-stage thymic tumors showing nuclear compositions of hetero- and euchromatin (scale bar representing 2μm). **(B)** Volcano plot depicting differential gene expression of biological replicates of NPM::ALK (n=4) and NPM::ALK *Hdac1*KO (n=4) end-stage thymic tumors based on RNA-seq analysis. Red represents significantly deregulated genes with |LFC| $\geq$ 1 and adj p < 0.05. **(C)** UCSC Genome Browser(1) ATAC-seq tracks depicting peaks, which represent open chromatin regions in *CD3d* and *Cd3g* genes. Biological replicates of NPM::ALK end-stage thymic tumors (n=4, blue) and of NPM::ALK *Hdac1*KO end-stage thymic tumors (n=4, red) are shown. Gencode tracks (Gencode VM23 release) below display corresponding transcripts. Colored boxes on the

bottom show ENCODE Candidate Cis-Regulatory Elements (cCREs) combined from all available cell types (red promoter, orange proximal enhancer, yellow distal enhancer).

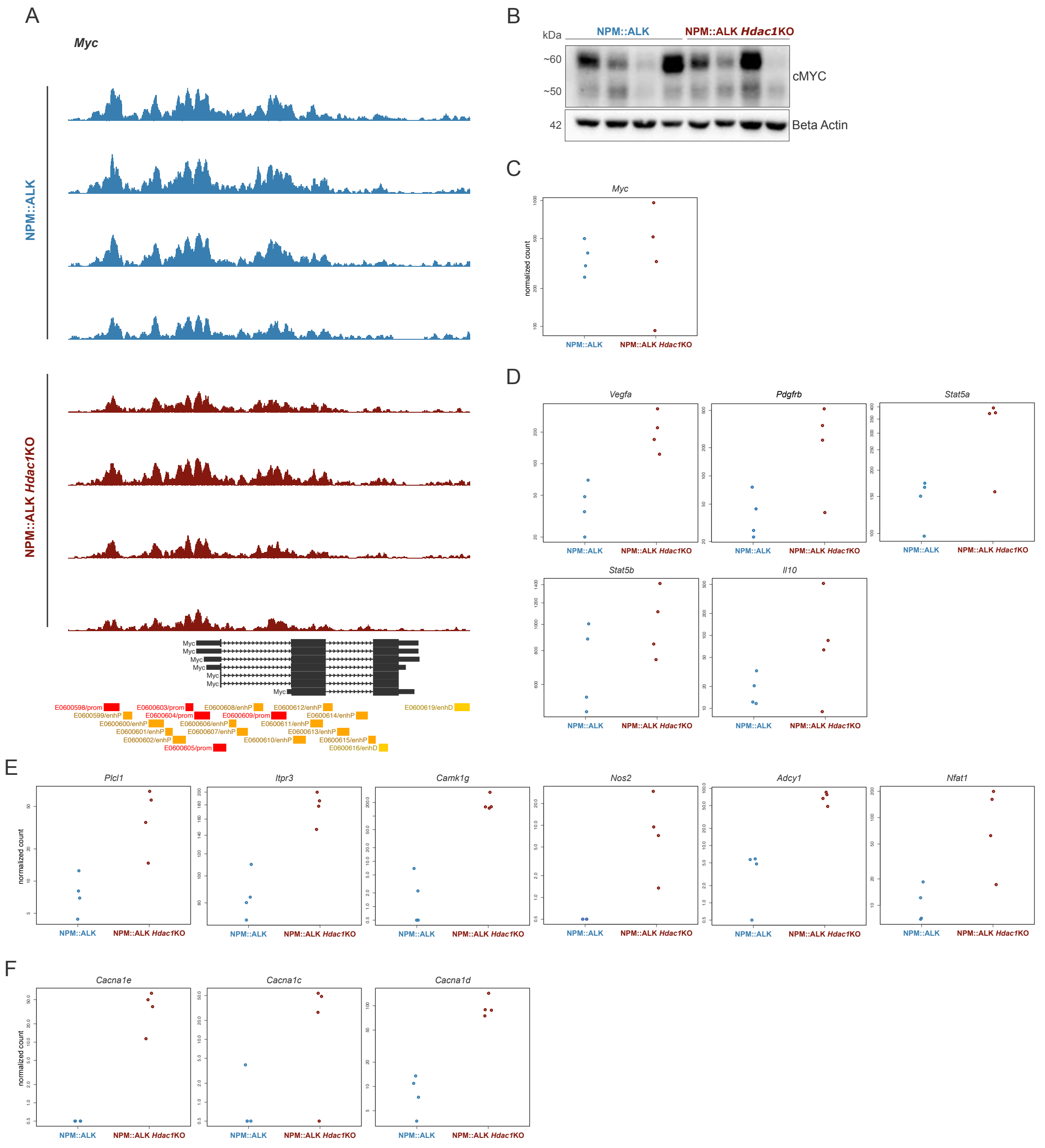

SUPPLEMENTAL FIGURE S6: Loss of *Hdac1* hyperactivates oncogenic transcription (A) UCSC Genome Browser(1) ATAC-seq tracks depicting peaks, which represent open chromatin regions in the *Myc* gene. Biological replicates of NPM::ALK end-stage thymic tumors (n=4, blue) and of NPM::ALK *Hdac1*KO end-stage thymic tumors (n=4, red) are shown. Gencode tracks (Gencode VM23 release) below display corresponding transcripts. Colored boxes on the bottom show ENCODE Candidate Cis-Regulatory Elements (cCREs) combined from all available cell types (red promoter, orange proximal enhancer, yellow distal enhancer). **(B)** Immunoblot showing protein expression levels of MYC in end-stage thymic tumors excised from NPM::ALK (n=4) and NPM::ALK *Hdac1*KO mice (n=4). Beta-Actin was used as a loading control. Numbers on the left indicate the molecular weight of analyzed proteins in kiloDalton (kDa). **(C)** VST normalized counts visualized from RNA-seq data for *Myc* comparing NPM::ALK (blue) and NPM::ALK *Hdac1*KO (red) samples. **(D)** VST normalized counts for *Vegfa, Pdgfrb, Stat5a, Stat5b* and *Il10* as in (C). **(E)** VST normalized counts for *Plcl1, Itpr3,*

Camk1g, Nos2, Adcy1 and *Nfat1* as in (C). **(F)** VST normalized counts for *Cacna1e, Cacna1c* and *Cacna1d* as in (C).

SUPPLEMENTAL TABLES

UPREGULATED GENES			
**Ingenuity Canonical Pathways**	**-log(p-value)**	**Ratio**	**Molecules**
Calcium-induced T Lymphocyte Apoptosis	3,69E+00	7,58E-02	CD3D,CD3G,CD4,HLA-A,ITPR3
OX40 Signaling Pathway	4,02E+00	6,67E-02	BCL2L1,CD3D,CD3G,CD4,HLA-A,NFKBIE
iCOS-iCOSL Signaling in T Helper Cells	4,45E+00	6,31E-02	CD3D,CD3G,CD4,HLA-A,ITPR3,NFKBIE,TRAT1
Regulation of IL-2 Expression in Activated and Anergic T Lymphocytes	3,07E+00	5,56E-02	CD3D,CD3G,NFKBIE,TGFBR1,VAV2
T Helper Cell Differentiation	2,52E+00	5,48E-02	HLA-A,TBX21,TGFBR1,TNFRSF1A
PKCŒ∏ Signaling in T Lymphocytes	4,38E+00	5,16E-02	CACNA1D,CACNG4,CD3D,CD3G,CD4,HLA-A,NFKBIE,VAV2
Th2 Pathway	3,89E+00	5,15E-02	CCR4,CD3D,CD3G,CD4,HLA-A,TBX21,TGFBR1
CD28 Signaling in T Helper Cells	3,32E+00	4,96E-02	CD3D,CD3G,CD4,HLA-A,ITPR3,NFKBIE
T Cell Receptor Signaling	2,75E+00	4,72E-02	CD3D,CD3G,CD4,PAG1,VAV2
Cdc42 Signaling	3,99E+00	4,55E-02	CD3D,CD3G,HLA-A,ITGA6,LIMK1,MYL10,TNK2,VAV2
CTLA4 Signaling in Cytotoxic T Lymphocytes	2,22E+00	4,49E-02	CD3D,CD3G,HLA-A,TRAT1
Death Receptor Signaling	2,16E+00	4,35E-02	BID,LIMK1,NFKBIE,TNFRSF1A
Th1 Pathway	2,50E+00	4,13E-02	CD3D,CD3G,CD4,HLA-A,TBX21
Th1 and Th2 Activation Pathway	3,28E+00	4,09E-02	CCR4,CD3D,CD3G,CD4,HLA-A,TBX21,TGFBR1
Apoptosis Signaling	2,04E+00	4,00E-02	BCL2L1,BID,NFKBIE,TNFRSF1A
Role of NFAT in Regulation of the Immune Response	3,14E+00	3,87E-02	CD3D,CD3G,CD4,HLA-A,ITPR3,NFKBIE,RCAN3
PD-1, PD-L1 cancer immunotherapy pathway	1,95E+00	3,77E-02	BCL2L1,HLA-A,PDCD1,TNFRSF1A
PI3K Signaling in B Lymphocytes	2,26E+00	3,62E-02	CARD10,IRS2,ITPR3,NFKBIE,VAV2
D-myo-inositol (1,4,5,6) / (3,4,5,6)-Tetrakisphosphate Biosynthesis	2,21E+00	3,52E-02	ALPL,DUSP10,PDCD1,PPP1R1B,PTPRF
3-phosphoinositide Degradation	2,04E+00	3,21E-02	ALPL,DUSP10,PDCD1,PPP1R1B,PTPRF
3-phosphoinositide Biosynthesis	1,93E+00	3,01E-02	ALPL,DUSP10,PDCD1,PPP1R1B,PTPRF
Calcium Signaling	2,16E+00	2,91E-02	CACNA1D,CACNG4,CAMK1G,CHRNA9,ITPR3,RCAN3
T Cell Exhaustion Signaling Pathway	1,84E+00	2,86E-02	HLA-A,PDCD1,TBX21,TGFBR1,VEGFA
Protein Kinase A Signaling	3,33E+00	2,75E-02	ADCY1,AKAP12,DUSP10,H1-0,ITPR3,MYL10,NFKBIE,PPP1R1B,PTPRF,TCF7L1,TGFBR1
Senescence Pathway	2,12E+00	2,55E-02	CACNA1D,CBX7,DHCR24,ETS2,ING1,ITPR3,TGFBR1
Phospholipase C Signaling	1,65E+00	2,26E-02	ADCY1,CD3D,CD3G,ITGA6,ITPR3,MYL10

Supplementary Table 1: Enriched pathways (upregulated genes)
Significantly enriched pathways in NPM::ALK *Hdac1*KO end-stage thymic tumors as compared to NPM::ALK end-stage thymic tumors based on Ingenuity Pathway Analysis (IPA®) of upregulated genes (RNA-seq: |LFC| $\geq$ 1, adj p < 0.05) that were correlated with changes in chromatin accessibility (correlation p < 0.5). P-value, represents significance of enrichment; ratio, indicates proportion of genes affected within each pathway, molecules, shows deregulated genes in each pathway.

DOWNREGULATED GENES			
**Ingenuity Canonical Pathways**	**-log(p-value)**	**Ratio**	**Molecules**
Wnt/β-catenin Signaling	4,64E+00	5,2E-02	ACVR1,CD44,FZD7,GJA1,HDAC1,SOX10,SOX8,TGFB2,WIF1
Regulation Of The Epithelial Mesenchymal Transition In Development Pathway	2,19E+00	4,76E-02	FZD7,PTCH1,PYGO2,SNAI2
Regulation of the Epithelial-Mesenchymal Transition Pathway	2,15E+00	3,12E-02	FGF7,FZD7,MAPK3,PYGO2,SNAI2,TGFB2
PFKFB4 Signaling Pathway	2,12E+00	6,52E-02	MAPK3,PRKAR2B,TGFB2
Protein Kinase A Signaling	2,05E+00	2,25E-02	AKAP6,EYA1,FLNB,GNB4,MAPK3,PPP1R3C,PRKAR2B,PTCH1,TGFB2
TGF-β Signaling	1,99E+00	4,17E-02	ACVR1,HDAC1,MAPK3,TGFB2
Gαs Signaling	1,83E+00	3,74E-02	GNB4,MAPK3,PRKAR2B,PTGER2
Glutathione Redox Reactions I	1,73E+00	8,33E-02	GPX7,GPX8
Role of JAK family kinases in IL-6-type Cytokine Signaling	1,70E+00	8,00E-02	IL6R,MAPK3
Signaling by Rho Family GTPases	1,61E+00	2,37E-02	CDC42EP3,CDC42EP5,CDH20,CYFIP1,GNB4,MAPK3
Ferroptosis Signaling Pathway	1,60E+00	3,17E-02	DPP4,MAPK3,SREBF2,TF
Regulation Of The Epithelial Mesenchymal Transition By Growth Factors Pathway	1,60E+00	2,66E-02	FGF7,IL6R,MAPK3,SNAI2,TGFB2
Sonic Hedgehog Signaling	1,58E+00	6,9E-02	PRKAR2B,PTCH1
STAT3 Pathway	1,51E+00	2,96E-02	IL6R,IL7R,MAPK3,TGFB2

Supplementary Table 2: Enriched pathways (downregulated genes)
Significantly enriched pathways in NPM::ALK *Hdac1*KO end-stage thymic tumors as compared to NPM::ALK end-stage thymic tumors based on Ingenuity Pathway Analysis (IPA®) of downregulated genes (RNA-seq: |LFC| $\geq$ 1, adj p < 0.05) that were correlated with changes in chromatin accessibility (correlation p < 0.5). P-value, represents significance of enrichment; ratio, indicates proportion of genes affected within each pathway, molecules, shows deregulated genes in each pathway.

REFERENCES

1. Raney BJ, Barber GP, Benet-Pagès A, Casper J, Clawson H, Cline MS, et al. The UCSC Genome Browser database: 2024 update. Nucleic Acids Res. 2023 Nov 11;52(D1):D1082–8.
